# Finite Element Analysis of a Bionate Ring-Shaped Customized Lumbar Disc Nucleus Prosthesis

**DOI:** 10.1021/acsabm.1c01027

**Published:** 2021-12-14

**Authors:** Amparo Vanaclocha-Saiz, Vicente Vanaclocha, Carlos M. Atienza, Pablo Clavel, Pablo Jorda-Gomez, Carlos Barrios, Leyre Vanaclocha

**Affiliations:** † Escuela de Doctorado, Universitat Politècnica de Valencia, Camí de Vera, s/n, 46022 Valencia, Spain; ‡ University of Valencia, Avenida de Blasco Ibáñez, 13, 46010 Valencia, Spain; § Instituto de Biomecánica (IBV), Universitat Politècnica de Valencia, Camí de Vera, s/n, 46022 Valencia. Spain; ∥ Instituto de Biomecánica de Valencia-CIBER BBN, Grupo de Tecnología Sanitaria (GTS-IBV), Camí de Vera, s/n, 46022 Valencia, Spain; ⊥ Instituto Clavel, Hospital Quironsalud Barcelona, Plaça d’Alfonso Comín, 5, 08023 Barcelona, Spain; # Hospital Politècnic i Universitari La Fe, Avinguda de Fernando Abril Martorell, 106, 46026 Valencia, Spain; ∇ Catholic University of Valencia, Saint Vincent Martyr, Carrer de Quevedo, 2, 46001 Valencia, Spain; ○ University College London, London, Gower St, London WC1E 6BT, U.K.

**Keywords:** degenerative disc disease, nucleus disc replacement, polycarbonate urethane, motion preservation, finite element model, disc hernia

## Abstract

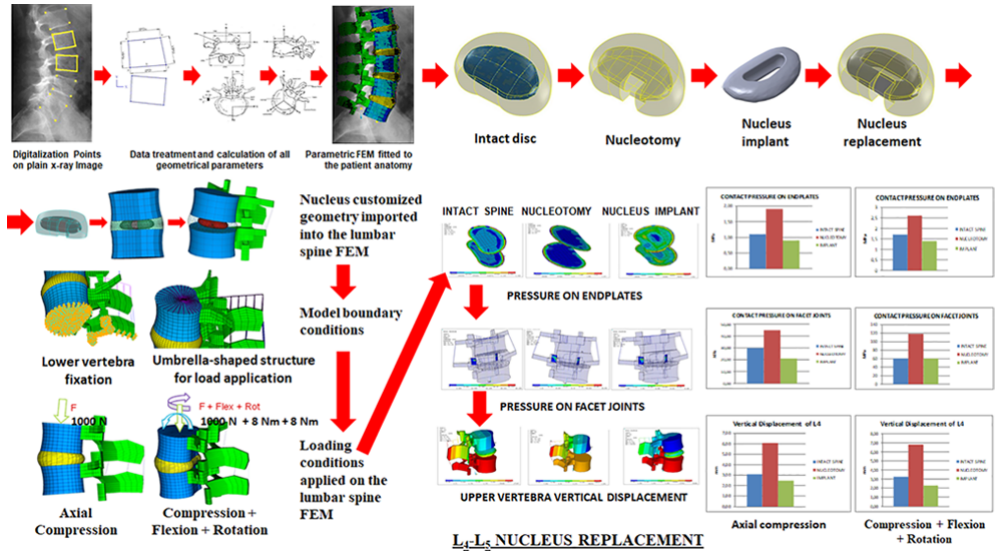

**Study design**: Biomechanical study of a nucleus replacement with a finite element model. **Objective**: To validate a Bionate 80A ring-shaped nucleus replacement. **Methods**: The ANSYS lumbar spine model made from lumbar spine X-rays and magnetic resonance images obtained from cadaveric spine specimens were used. All materials were assumed homogeneous, isotropic, and linearly elastic. We studied three options: intact spine, nucleotomy, and nucleus implant. Two loading conditions were evaluated at L_3_-L_4_, L_4_-L_5_, and L_5_-S_1_ discs: a 1000 N axial compression load and this load after the addition of 8 Nm flexion moment in the sagittal plane plus 8 Nm axial rotation torque. **Results**: Maximum nucleus implant axial compression stresses in the range of 16–34 MPa and tensile stress in the range of 5–16 MPa, below Bionate 80A resistance were obtained. Therefore, there is little risk of permanent implant deformation or severe damage under normal loading conditions. Nucleotomy increased segment mobility, zygapophyseal joint and end plate pressures, and annulus stresses and strains. All these parameters were restored satisfactorily by nucleus replacement but never reached the intact status. In addition, annulus stresses and strains were lower with the nucleus implant than in the intact spine under axial compression and higher under complex loading conditions. **Conclusions**: Under normal loading conditions, there is a negligible risk of nucleus replacement, permanent deformation or severe damage. Nucleotomy increased segmental mobility, zygapophyseal joint pressures, and annulus stresses and strains. Nucleus replacement restored segmental mobility and zygapophyseal joint pressures close to the intact spine. End plate pressures were similar for the intact and nucleus implant conditions under both loading modes. Manufacturing customized nucleus implants is considered feasible, as satisfactory biomechanical performance is confirmed.

## 1 Introduction

Lumbar back pain is one of the most common diseases in modern sedentary society.^1^ Although its etiology is ample,^2^ degenerative disc disease and disc herniation are leading causes.^3^ Surgical treatments for these entities can be divided into fusion and motion preservation.^4^ Among the latter, we found total disc prosthesis^5^ and nucleus replacement to be suitable.^6^ The second is mainly indicated for disc herniation and early disc degeneration with a preserved annulus fibrosus,^6^ while total disc prosthesis is recommended in severe disc disease.^7^

Many nucleus disc replacements have been designed in the past, with only a few reaching the market^8^ and even less still in clinical use. The problems are varied and include material degradation,^9^ design flaws, extrusion,^10^ and subsidence^11^—the search for the ideal nucleus replacement material and design continues.^12^ Therefore, we decided to create a new nucleus implant based on past issues and failures.

The first step was selecting the material for the nucleus replacement, and the second was to make a suitable design. In earlier studies, we already took both steps. This article will analyze our new nucleus replacement properties and characteristics through a finite element model (FEM). This methodology allows implant design evaluation before manufacturing, cost savings, design improvement, and future optimizations.^13^ It has limitations as a computer simulation study but it is easy to use and mimics different clinical scenarios.^14^

We aimed to assess with a lumbar spine parametric FEM a new ring-shaped nucleus implant made of a polymeric material (Bionate 80A, The Polymer Technology Group DSM-PTG, Berkeley, California).^15^ Under different loading conditions, we analyzed implant mechanical responses and interactions between operated discs and surrounding anatomical structures, such as spinal ligaments, annulus, end plates, and facet joints. In addition, a complete biomechanical analysis was performed with the lumbar spine FEM on customized nucleus implants to assess their functionality and feasibility. To do it, we used a lumbar spine FEM model previously developed by the IBV (Institute of Biomechanics of Valencia, Valencia, Spain)^16^ that allows customization and reproduction of any specific patient lumbar spine anatomy. In addition, the model has other adjustable features like tissue mechanical properties or mesh density and can be changed to reproduce surgical procedures like nucleotomy, annulotomy, or nucleus replacement. The results of this study will be presented here.

## 2 Materials and Methods

### 2.1 Biomechanical Evaluation Protocol

**Figure 1:**
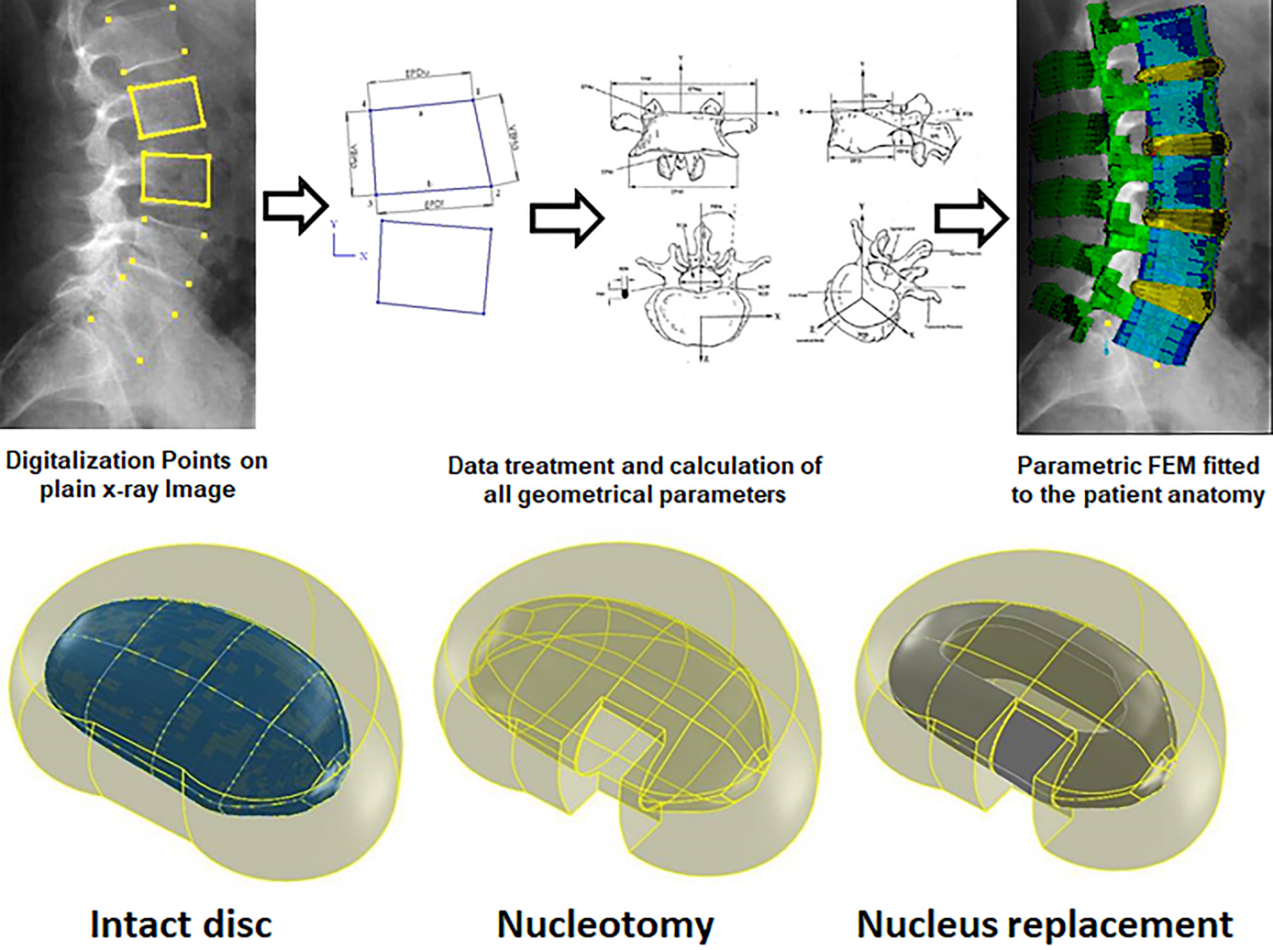
Above, the process for customization and generation of lumbar spine FEM. Below, different disc model configurations were obtained with the CAD software (SolidWorks).

The IBV lumbar spine model is parametric and programmed in Ansys Parametric Design Language (APDL), allowing geometrical customization and reproducing patient lumbar spine three-dimensional (3D) geometry from a small set of parameters. In addition, specialized software named orthoCapture was used. We used lumbar spine sagittal plane X-ray and magnetic resonance imaging (MRI) images to digitalize the four points defining each vertebral body limits, obtaining vertebral body height and depth, and vertebrae layout. The output contained the two-dimensional (2D) coordinates of the points defining vertebral body limits (Figure 1, above). The primary input for the FEM software (ANSYS) lumbar spine model geometrical generation was these coordinate files. The other geometrical parameters to build the model were calculated from these initial sagittal parameters and geometrical relationships derived from different published studies.^14,17,18^ The mesh density was crucial because the thicker the density the more accurate the calculations were, and the computation time was also longer.

The model generation is automatically defined in ANSYS implant and different anatomical structure properties. Therefore, they could be modified, but as their values are usually the same, it was considered more efficient to define defect values and change them when any material property modification was made. All materials were assumed to be homogeneous, isotropic, and linearly elastic, and their characteristics were collected from the literature.^19−34^

We studied three different options: intact spine, nucleotomy, and nucleus implant. Since the customized nucleus had a complex geometry, it could not be defined within the program; it was done outside with CAD software and then imported into the spine FEM. For building the intervertebral disc outside the model, a file containing the coordinates of the main disc dimensions was exported from ANSYS. We used this coordinates file to generate the disc 3D geometry with CAD software (SolidWorks, Dassault Systèmes, Vélizy-Villacoublay, France). Other essential inputs for recreating the treated disc were the CAD files with the original nucleus and the customized nucleus implant geometries.

Three different 3D disc models were created (Figure 1, lower panel). The first was the intact disc, with the nucleus pulposus inside the disc geometry in the same position and orientation as in axial MRI images. In the second configuration, the nucleotomy, a cavity was created to reproduce nucleus removal, and a posterior annulotomy was added. In the third configuration, the nucleus replacement, a customized implant was placed in the correct position inside the nucleus cavity. The material selected was Bionate 80A, with *E* = 22.19–23.93 MPa; *v*(Poisson coefficient) = 0.4923–0.4924^35^ and elastic modulus = 22 MPa^35^ and with a hollow compressible monobloc elastomeric design with a 5 mm wall (Figure 1). As in the nucleotomy, a posterior annulotomy defect was simulated. The final disc had the same geometry as the parametric lumbar spine model, with the only difference that the nucleus shape and volume was not parametric but customized. The studies were repeated for the L_3_-L_4_, L_4_-L_5_, and L_5_-S_1_ discs, as each has peculiar characteristics. For example, L_5_-S_1_ discs have to support a higher shear force than the other two,^36^ and the zygapophyseal joint shape and orientation are different for all of them.^37^

**Figure 2:**
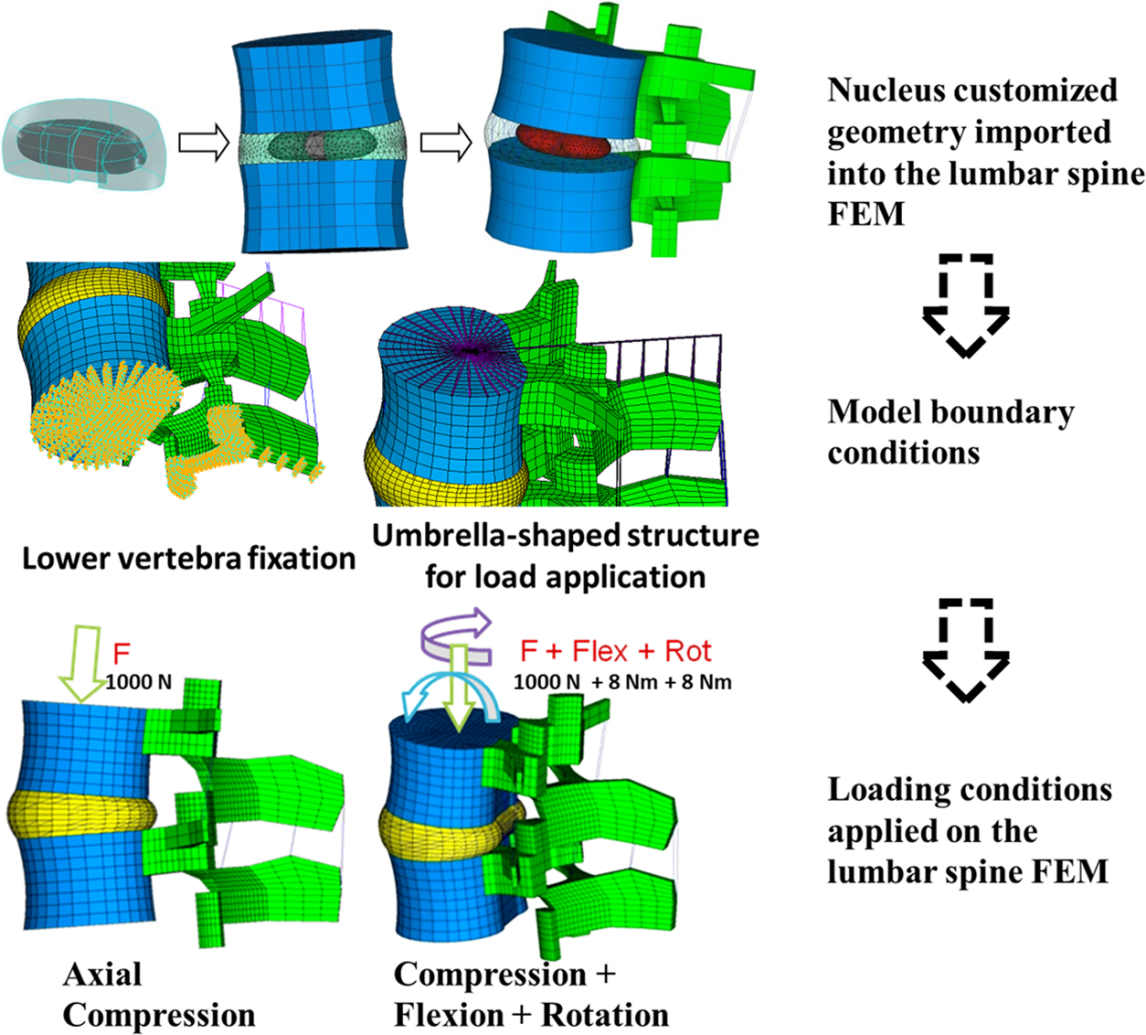
Finite element model process.

Once the final disc configuration had been built with CAD software, the treated disc was imported in ANSYS into the lumbar spine FEM (finite element model), replacing the old untreated disc (Figure 2). The new disc was meshed and joined to the adjacent vertebras, and free meshing was applied with four-node solid elements. The disc and annulus fibers were assigned the mechanical properties reported in earlier models.^38^ The interfaces between disc and vertebral end plates were defined with bonded contact elements. The interface between implant and annulus in the nucleus implant configuration was modeled with contact elements with a friction coefficient of 0.02. The entire process was programmed and integrated with the rest of the model, making it possible to change the nucleus mesh density and properties of the material.

Once the definitive FEM was built, the last step before calculation was defining the loading and boundary conditions. We performed FEA compression (BS ISO 7743:2008) and shear modulus tests (BS ISO 1827:2007). We applied 1000 N axial compression (like a spinal load when walking), 1000 N axial compression N plus 300 N anteroposterior shear, and 1000 N axial compression with 8 Nm sagittal plane flexion moment plus axial 8 Nm rotation torque (the scenario with a high disc herniation risk). Nodes below the lower vertebra were fixed for load application, displacements, and rotations; the nodes in the upper edge of the upper vertebra joined the superior central node of the same vertebra with link elements, and the nodes of the spinous process merged with the previous structure also with link elements. The load was spread evenly within this structure and placed in the upper vertebra’s top central node.

The boundary conditions were always the same, although they could be modified at will. The inferior surfaces of the inferior vertebra were ultimately constrained, and the loads applied on an umbrella-shaped structure fixed over the superior surface of the upper vertebra (Figure 2). The load was spread evenly with this structure and placed in the upper vertebra top central node. This node was also the central one of the above-described structure.

Two different loading conditions were considered. A 1000 N axial compression load was applied in the first loading condition, typical of lumbar spine normal daily activities (i.e., walking). In the second, an 8 Nm flexion moment in the sagittal plane with 8 Nm axial rotation torque was added to the 1000 N axial load, representing the worst-case scenario with a high potential for producing disc herniation or nucleus implant expulsion (Figure 2).

Numerical computing took place once everything had been defined. The customized nucleus replacement biomechanical analysis parameters were implant stresses, inner annulus stresses and strains, end plate contact pressures, facet joint contact pressures, and relative displacements between vertebrae. Implant stresses revealed implant performance and endurance, and inner annulus stresses and strains clarified implant load transmission to annulus inner layers. End plate contact pressures correlated with the implant subsidence risk. Zygapophyseal joint contact pressures were critical because overloading them may induce degenerative changes. Finally, relative displacements between vertebrae allowed implant performance and flexibility comparisons between operated and intact spines.

The mechanical results from implanted and intact vertebral segments were compared, and depending on how far from each other were both results, customized implant design was considered acceptable or not. In addition, the nucleotomy data were compared with the intact spine since this is a usual surgical alternative for herniated discs.

### 2.2 Cadaveric Lumbar Spine Biomechanical Evaluation

Six cadaveric lumbar spines supplied the *Facultat de Medicina i Odontologia*, University of Valencia, Spain, cold preserved since demise, were chosen for biomechanical evaluation. Muscles and other soft tissues were removed, keeping ligaments and intervertebral discs intact and spines sectioned on T_12_-L_1_ intervertebral disc and sacroiliac joints. To be eligible, they should not have had any earlier lumbosacral spine surgical procedure, traumatism, or oncologic, infectious, or inflammatory disease. Plain X-ray studies and dual energy X-ray absorptiometry (DEXA) scans were done to rule out osteoporosis. Additionally, every cadaveric spine specimen underwent an MRI to obtain its geometry to design the customized nucleus implant.

All cadaveric spines underwent the biomechanical evaluation protocol described in the section above. In addition, different FEM scenarios were considered: intact spine, nucleotomy, and nucleus implant, and the same loading conditions simulated in every case for the L_3_-L_4_, L_4_-L_5_, and L_5_-S_1_ discs. Finally, their results were compared to find the nucleus replacement biomechanical results and the differences between the intact and the customized nucleus replacement lumbar spines.

## 3 Statistical Analysis

We used Excel (Microsoft Corporation, Redmond, WA) and SPSS 26 (IBM Corporation, Armonk, New York, US) for data analysis, and we calculated movement angles and parameters using GNU Octave software (GNU General Public License, https://www.gnu.org/software/octave/index). In addition, the statistical analysis R (R Development Core Team; Kirby and Gerlanc, 2013; R: The R Project for Statistical Computing, n.d.^39^) and the Deducer user interface (I. Fellows, Deducer: A Data Analysis GUI for R, Journal of Statistical Software, vol. 49, No. 8, 2012.)^40^ were also used in combination.

## 4 Results

The results and conclusions for each disc and condition (intact spine, nucleotomy, and nucleus replacement) are presented next. Further detailed graphical results for every mechanical parameter considered for the study are shown in the Supporting Material.

### 4.1 L_3_-L_4_ Nucleus Replacement

**Figure 3:**
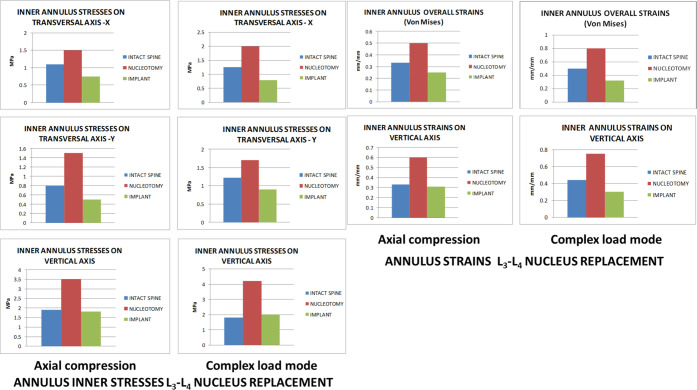
Annulus inner stresses and strains in the L_3_-L_4_ disc after nucleus replacement.

In both loading modes, maximum nucleus implant stresses were 22 MPa for compression stress and 5 MPa for tensile stress, both values below the nucleus implant material (50 MPa for compression stress and 47 MPa for tensile stress). According to these results, there was no risk of nucleus implant permanent deformation or severe damage under normal loading conditions. Nucleotomy increased the mobility under both loading conditions, with a considerable augmentation in facet joint pressure and annulus stresses and strains. Nucleus replacement restored the mobility altered by nucleotomy, making it closer to the intact spine, but was slightly lower than the original intact spine in the single-axial compression mode. In contrast, it was higher in the complex load mode than in the original untouched state (Figure 3).

**Figure 4:**
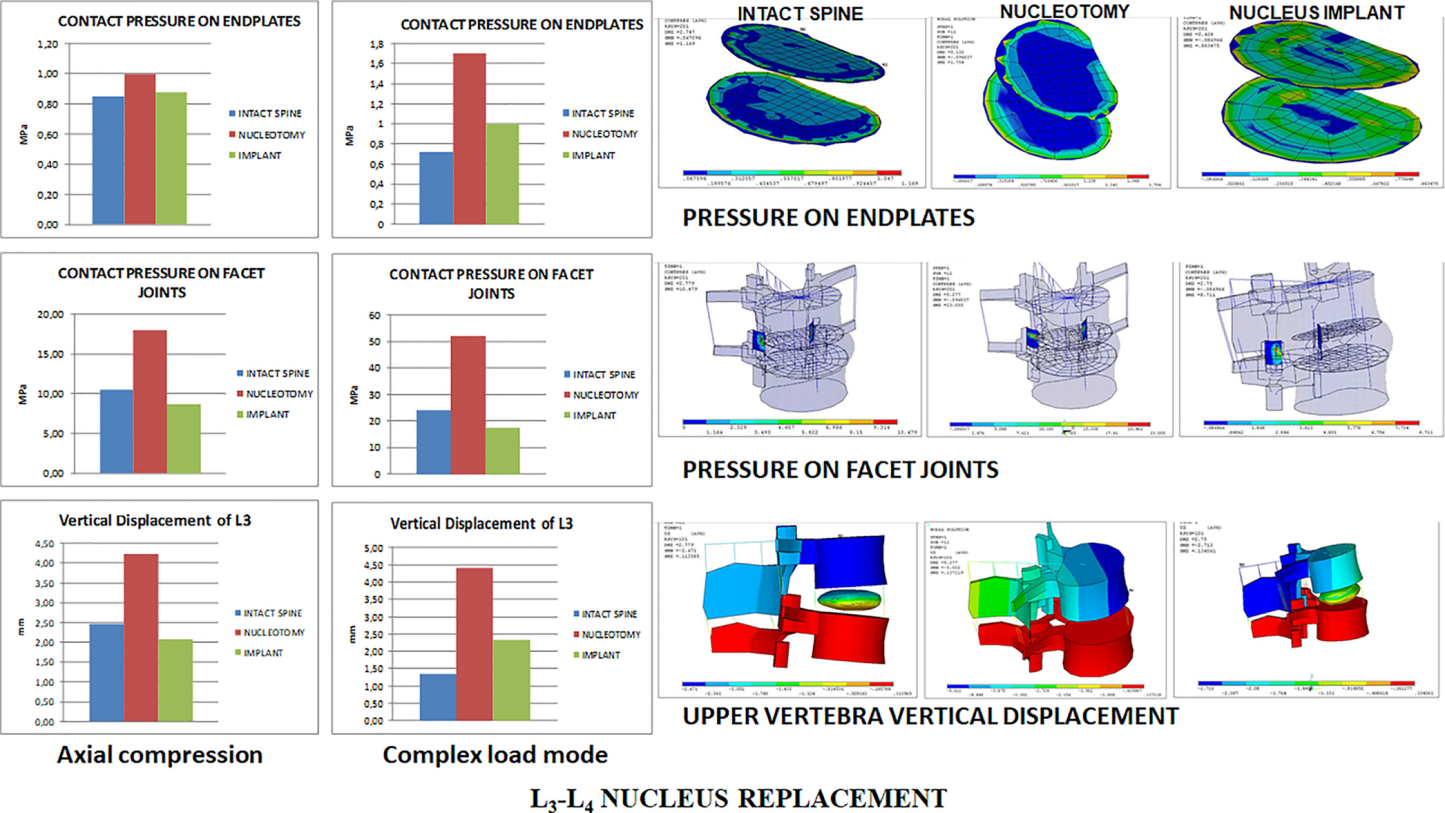
L_3_-L_4_ facet joint and end plate pressures and upper vertebra vertical displacement.

It also restored facet joint pressures slightly below the intact condition (Figure 4) and annulus stresses and strains on the axial compression and complex load modes. However, stresses and strains transmitted on the transversal plane to the inner annulus were lower with the nucleus implant than in the intact spine. Pressures on end plates were similar between the unoperated and nucleus implant states under both loading modes, while nucleotomy produced higher pressures under complex loading conditions (Figure 4).

Further information is provided in supporting material Figures 1S–8S.

### 4.2 L_4_-L_5_ Nucleus Replacement

**Figure 5:**
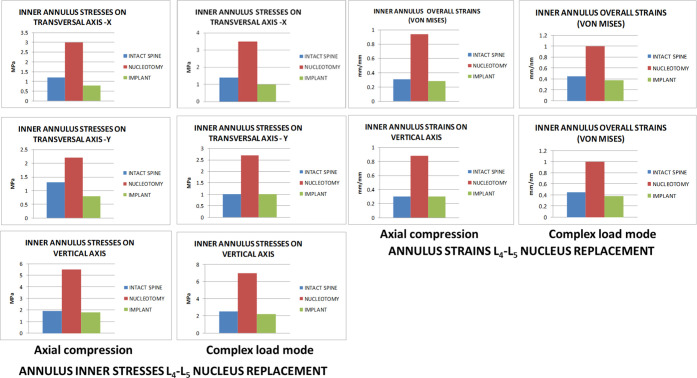
Annulus inner stresses and strains in the L_4_-L_5_ disc after nucleus replacement.

The maximum stresses on the nucleus implant were 34 MPa for the compression stress and 16 MPa for the tensile stress in both loading modes. As both values are below the strength limits of the nucleus implant material, as mentioned above, no risk of nucleus replacement permanent deformation or severe damage was expected under normal loading conditions. Nucleotomy increased mobility under loading conditions and considerable augmentation in facet joint and end plate contact pressures and annulus stresses and strains. Nucleus implant restored the mobility altered by nucleotomy, making it closer but slightly lower than the intact spine (Figure 5).

**Figure 6:**
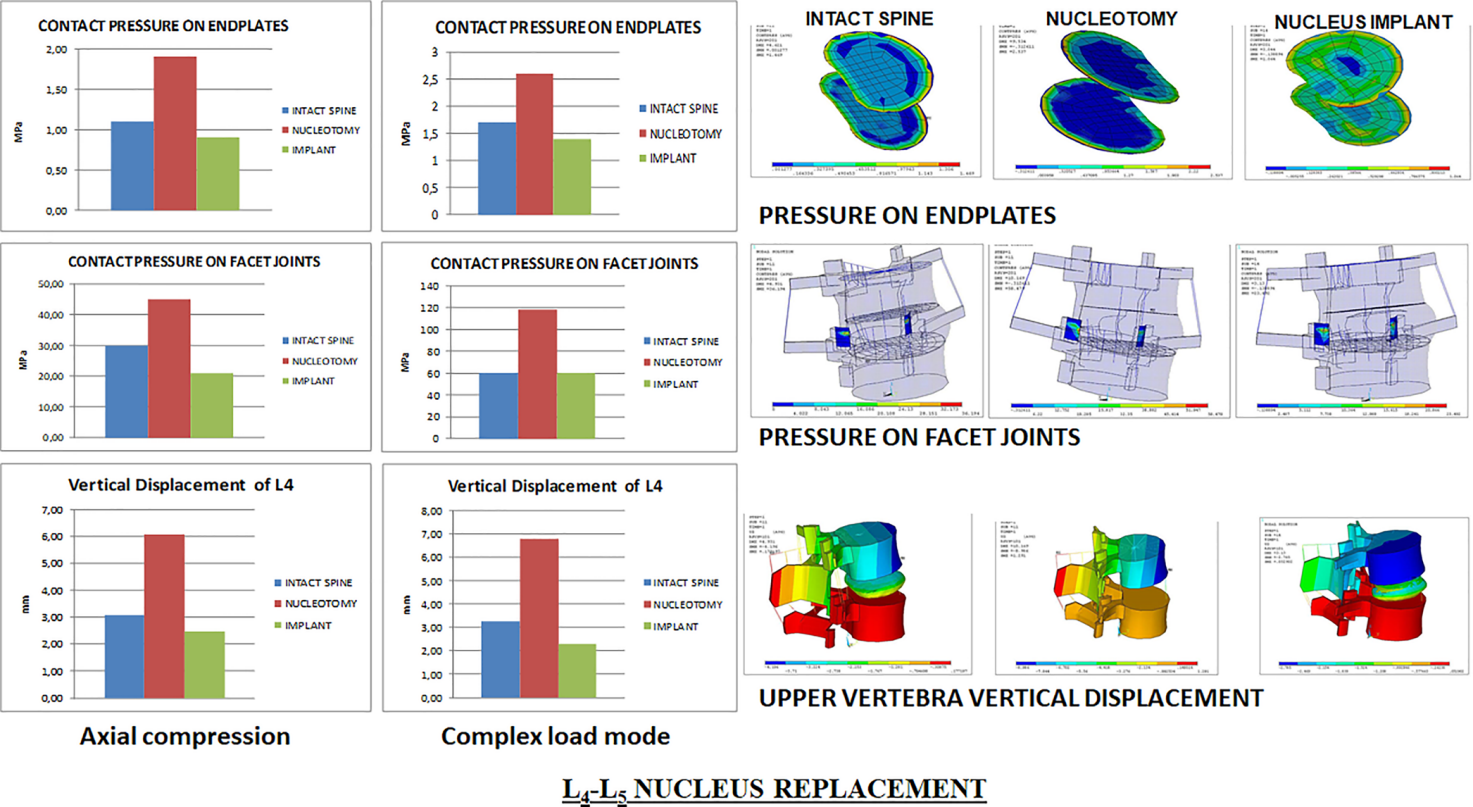
L_4_-L_5_ facet joint and end plate pressures and upper vertebra vertical displacement.

It recovered facet joint and end plate pressures and annulus stresses and strains under axial compression and complex loadings, but stresses and strains transmitted to the transversal plane inner annulus were lower than those in the intact spine but still better than with nucleotomy (Figure 6).

Further information is provided in supporting material Figures 9S–16S.

### 4.3 L_5_-S_1_ Nucleus Replacement

**Figure 7:**
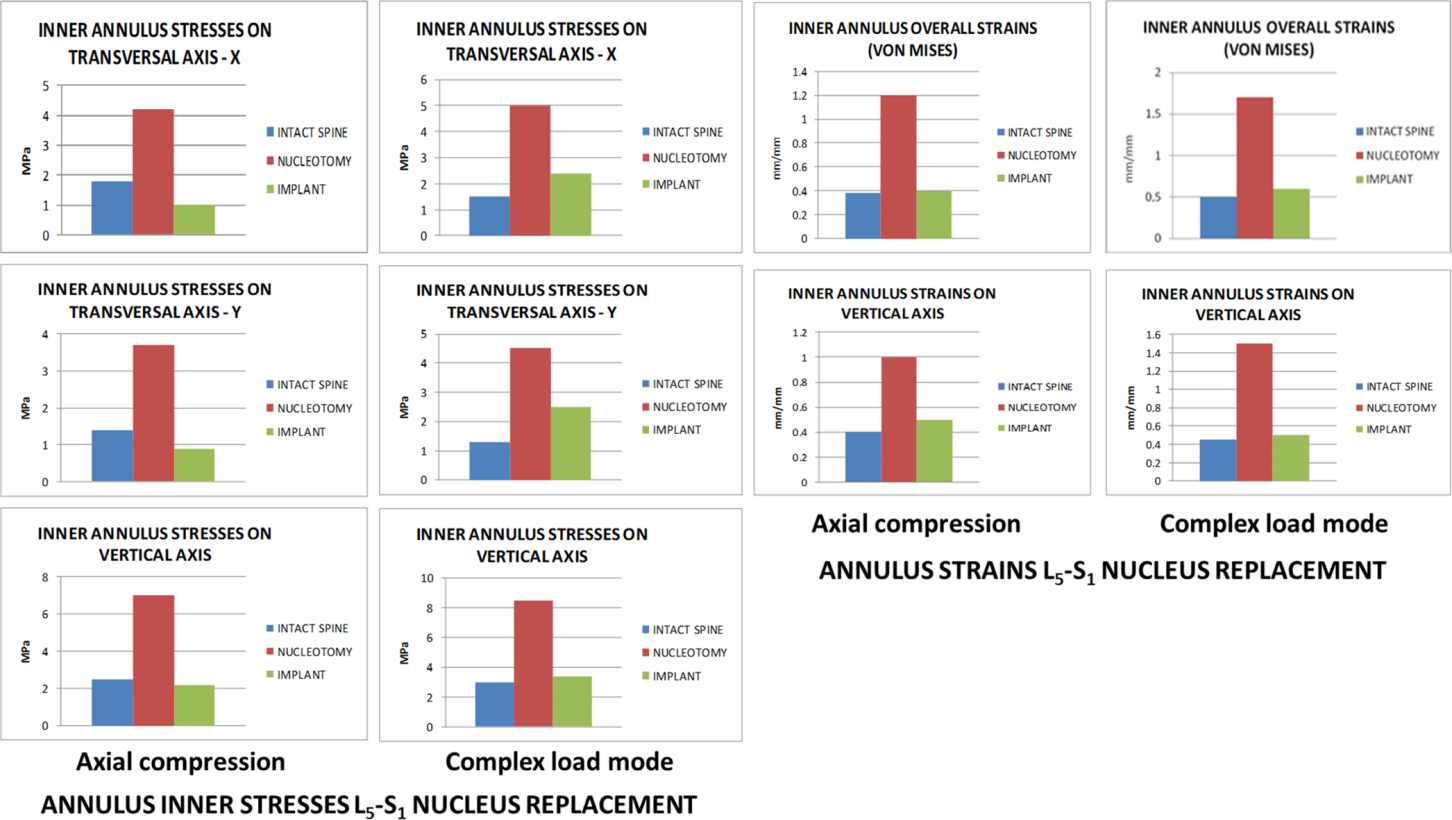
Annulus inner stresses and strains in the L_5_-S_1_ disc after nucleus replacement.

Under axial compression, the maximum stresses on the nucleus implant were 25 MPa for the compression stress and 10 MPa for the tensile stress. As both values were below the strength limits of the nucleus implant material, under normal loading conditions, there was no risk of permanent deformation or severe damage. However, nucleotomy increased mobility under both loading conditions with a considerable augmentation in facet joint pressures, especially under complex loading conditions and increased stresses and strains in the annulus (Figure 7).

**Figure 8:**
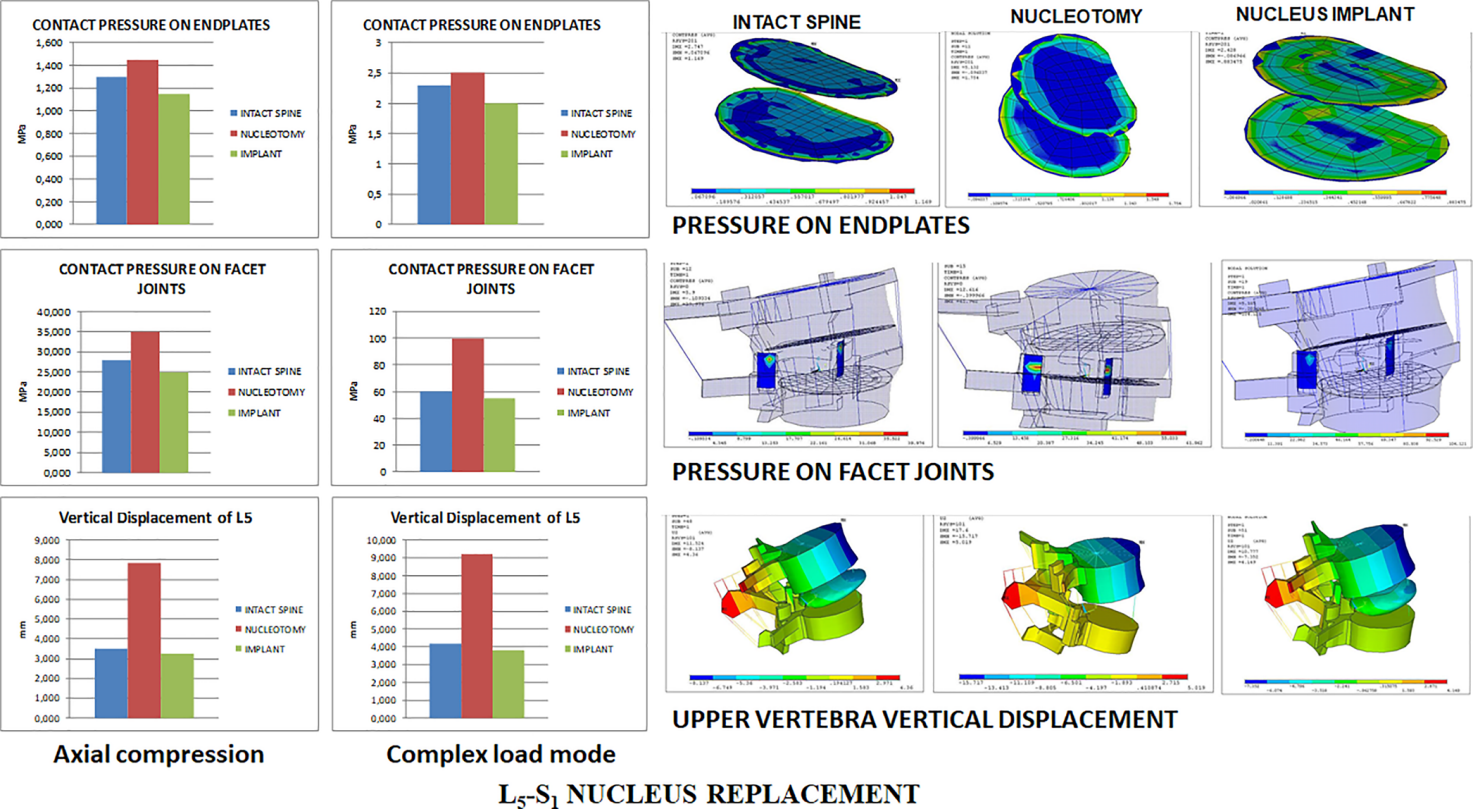
L_5_-S_1_ facet joint and end plate pressures and upper vertebra vertical displacement.

The nucleus implant restored the lumbar segment mobility, facet joint pressures, and annulus stresses and strains under both loading conditions (Figure 8). Under single-axial compression, nucleus replacement stress and strain transmission to the inner annulus in the transversal plane was lower than in the intact spine and higher under complex loading conditions. Compared to nucleotomy, stresses and strains transmitted to the annulus with the nucleus implant were closer to the intact spine. Pressures on end plates were remarkably similar among the three scenarios: natural state, nucleotomy, and nucleus implant (Figure 8).

Further information is provided in supporting material Figures 18S–24S.

### 4.4 Summary of L_3_-L_4_, L_4_-L_5_, and L_5_-S_1_ Intact, Nucleotomy, and Nucleus Replacjement FEA

The maximum nucleus implant axial compression stresses were 16 (L_5_-S_1_), 25 (L_3_-L_4_), and 34 MPa (L_4_-L_5_) as well as tensile stresses were 5 (L_3_-L_4_), 10 (L_5_-S_1_), and 16 MPa (L_4_-L_5_). As nucleus implant strength limits were 50 MPa for the compression stress and 47 MPa for the tensile stress, it had no risk of permanent deformation or severe damage under normal loading conditions.

Nucleotomy increased segment mobility under loading conditions in all three studied discs, inducing a considerable augmentation in facet joint pressures, increased annulus stresses and strains, and in L_5_-S_1_, especially under complex loading conditions.

Nucleus implant restored the lumbar segment mobility altered by nucleotomy, achieving mobility close to the intact spine in all three studied discs. In L_3_-L_4_, it was slightly lower in the single-axial compression mode than in the original intact spine, whereas in the complex load mode, it was higher than the original intact state. In L_4_-L_5_, disc mobility was slightly lower than the original untouched segment under both loading conditions. In L_5_-S_1_, mobility had remarkably similar values to that of the original intact segment under both loading conditions.

Nucleus replacement restored facet joint pressures in L_4_-L_5_ and L_5_-S_1_, while in L_3_-L_4_ they were slightly below the intact state. It also recovered annulus stresses and strains on the vertical axial compression and complex load modes (L_3_-L_4_ and L_4_-L_5_), mainly in the vertical axis (L_5_-S_1_). Compared to nucleotomy, stresses and strains transmitted to the annulus with the nucleus implant are closer to the intact spine, particularly at L_4_-L_5_ and L_5_-S_1_. However, in all discs under single-axial compression, stresses and strains transmitted to the transversal plane inner annulus were lower with the nucleus implant than in the intact spine, and under complex loading conditions, stress and strain transmission were higher than those in the intact case.

Pressures on end plates were similar between the unoperated and nucleus implant states under both loading modes. Nucleotomy produced higher pressures under complex loading conditions for L_3_-L_4_ and L_4_-L_5_ discs, but L_5_-S_1_ were remarkably similar for the natural state, nucleotomy, and nucleus implant.

Customized nucleus implants showed a good overall biomechanical performance in all three studies discs, and thus, manufacturing was deemed feasible.

## 5 Discussion

Nucleotomy is the current treatment for disc hernia, particularly in the lumbar spine. From the biomechanical point of view, it is known to induce biomechanical instability,^41^ reduce disk height,^42^ increase segmental mobility,^43^ and, consequently, abnormal annulus stress distribution^43^ and acceleration of zygapophyseal joint degeneration.^44^ Although patients do well initially, in the mid to long term, they start to notice chronic low back pain^45^ that eventually radiates to one or both lower limbs.^46^ Physiotherapy and muscle strengthening exercises are helpful^47^ until symptoms get so severe that a spinal fusion must be considered.^48^

The fundamental question is: will a nucleus replacement inserted in the index surgical procedure to remove the extruded disc recover the biomechanical characteristics of the disc^49^ and change this slow but inevitable path?^50^

Over the years, there have been many attempts in this arena. One of the most significant was the PDN nucleus implant, introduced by Ray.^51^ The basic concept was to use a material that would swell up once inserted and recover the disc height and mobility. However, sadly, problems arose among other reasons due to excessive implant rigidity upon complete swelling after being implanted inside the discal space.^6^ There were some cases of implant migration even with extrusion^10,52−54^ with radicular damage that, in some unfortunate cases, ended up in a cauda equina syndrome. Numerous attempts have been made ever since,^54,55^ and many companies have invested vast amounts of money in finding the perfect implant. The ways are varied, implants aiming to restore and regenerate the cellular nucleus pulposus, hydrogels,^43,56^ polymeric biomaterials,^54,57^ polyurethane,^58^ carboxymethylcellulose,^59^ graphite covered with pyrolytic carbon,^60^ and even an articulated nucleus resembling more a total disc replacement than an actual nucleus pulposus.^61^ Only a few of these prototypes have reached the market,^6,51,60,62^ and most, if not all, are now of very limited or no clinical use. The nucleus replacements fail particularly in bending and torsion under physiological loads.^63^ Is there any hope to find a solution to this problem or should we abandon the idea altogether?^12^

We present here a new attempt to solve an old problem. Our study concludes that the nucleus replacement improves the status compared to nucleotomy but our implant does not recover all baseline conditions completely. Values are better than with nucleotomy but still not quite the same as the intact disc. These results are similar to those reported in other nucleus replacements made of different materials like collagen combined with an annulus closure device,^41^ polyurethane (Newcleus, Sulzer Medica Inc, Switzerland)^64^ hydrogel,^42^ injectable cellulose-based hydrogel,^59^ and poly(vinylpyrrolidone) hydrogel.^43^ The results from our studies support that BIONATE is relevant for use in this kind of prosthesis with an elastic modulus of 22 MPa, more similar to the vertebral body cortical bone (14.64 MPa)^65^ with a Poisson ratio of 0.49,^35^ closer to the one for the nucleus pulposus (0.40).

Studying the characteristics and biomechanical results of our nucleus implant with FEM and the responses induced in nearby anatomical structures has been very useful to validate it, confirming the results obtained by other research groups with this same research tool.^65−67^ The selected material seems to stand the needed biomechanical requirements with a negligible risk of permanent deformation or severe damage. The design appears to minimize the chances of subsidence or extrusion, and the central cavity seems to buffer axial compression loads, as reported by similarly shaped nucleus implants.^66^ The zygapophyseal joint and end plate pressures seem to recover sufficiently, but the transversal axis (*x*- and *y*-axes) stresses on the annulus do not recover as well as desired. A compact design with the same material would probably solve this problem, but this would be at the price of higher subsidence and extrusion risk, as already seen in other designs.^52,68,69^ It seems that empty central space is the best way to provide a buffering effect to allow some controlled implant deformation on loading.^70^

However, this is a computer-generated reproduction. The data are fascinating and show us the flaws and ways of further improvement. It is necessary to prospect the situation and avoid unnecessary costs, particularly in this economically depressed era. We need studies with manufactured prototypes implanted in cadaveric spines. These studies should show light and help us decide if more steps are reasonable or we should, like many others, abandon the quest to find a nucleus replacement.

## 6 Limitations

The study is a computer simulation and not an actual clinical scenario, and data obtained are short-term and acute. The number of cadaveric spine specimens used to gather anatomical data is limited. Long-term fatigue and wear studies have not been done yet. Studies on nucleus implants inserted on cadaveric spines are needed to confirm the data obtained.

## 7 Strengths

FEM studies have repeatedly been shown to correlate with the results obtained with cadaveric spines. Additionally, an axial compression load and complex load mode with the same compression load but adding flexion in the sagittal plane and axial torsion were considered. The amount of data is vast and allows validation and improvements in nucleus replacement design and material choice.

## 8 Conclusions

The lumbar spine parametric FEM can reproduce any specific lumbar spine anatomy and confirm any new nucleus implant, evaluating the mechanical response under different loading conditions of adjacent anatomical structures like annulus, vertebral end plates, and zygapophyseal joints.

The maximum nucleus replacement compression and tensile stress values were below the nucleus implant material (Bionate 80A). Therefore, under normal loading conditions, there was no risk of permanent deformation or severe damage.

Nucleotomy increased segmental mobility under both loading conditions, augmenting considerably zygapophyseal joint pressures and annulus stresses and strains, especially under complex loading conditions.

Disc nucleus replacement restored segmental mobility and zygapophyseal joint pressures increased by nucleotomy, with values close to the intact spine. The *z*-axis also recovered annulus stresses and strains on both loading modes, but axial compression in the *x-* and *y*-axis was lower and under complex loading conditions higher than in the intact state.

End plate pressures were similar for intact and nucleus implant states under both loading modes. Nucleotomy produced higher end plate pressures under complex loading conditions for L_3_-L_4_ and L_4_-L_5_; however, for L_5_-S_1,_ no statistically significant differences were seen between the natural state, nucleotomy, and nucleus implant.

Customized nucleus implants showed a satisfactory overall biomechanical performance in all discs. Therefore, manufacturing was considered feasible.

## Supporting Information Available

The authors have provided supporting material as images that are detailed at the end of this manuscript; Figure 1S. L3-L4 nucleus replacement implant maximum stresses; Figure 2S. L3-L4 nucleus replacement annulus stresses; Figure 3S. L3-L4 nucleus replacement annulus strains under axial compression mode; Figure 4S. L3-L4 nucleus replacement annulus stresses; Figure 5S. L3-L4 nucleus replacement annulus strains under complex load mode; Figure 6S. L3-L4 nucleus replacement annulus Von Misses strains; Figure 7S. L3-L4 nucleus replacement relative motion of vertebrae under axial compression mode; Figure 8S. L3-L4 nucleus replacement relative motion of vertebrae under complex load mode; Figure 9S. L4-L5 nucleus replacement implant maximum stresses; Figure 10S. L4-L5 nucleus replacement annulus stresses under axial compression mode; Figure 11S. L4-L5 nucleus replacement annulus strains under axial compression mode; Figure 12S. L4-L5 nucleus replacement annulus stresses under complex load mode; Figure 13S. L4-L5 nucleus replacement annulus strains under complex load mode; Figure 14S. L4-L5 nucleus replacement annulus Von Misses strains; Figure 15S. L4-L5 nucleus replacement relative motion of vertebrae under axial compression mode; Figure 16S. L4-L5 nucleus replacement relative motion of vertebrae under complex load mode; Figure 17S. L5-S1 nucleus replacement implant maximum stresses under axial compression and complex load modes; Figure 18S. L5-S1 nucleus replacement annulus stresses under axial compression mode; Figure 19S. L5-S1 nucleus replacement annulus strains under axial compression mode; Figure 20S. L5-S1 nucleus replacement annulus stresses under complex load mode; Figure 21S. L5-S1 nucleus replacement annulus strains under complex load mode; Figure 22S. L5-S1 nucleus replacement annulus Von Misses strains under axial compression and complex load modes; Figure 23S. L5-S1 nucleus replacement relative motion of vertebrae under axial compression mode; and Figure 24S. L5-S1 nucleus replacement relative motion of vertebrae under complex load mode (PDF)
