# Supplementary material for: Finite Element Analysis of a Bionate Ring-Shaped Customized Lumbar Disc Nucleus Prosthesis
Source: ACS Appl Bio Mater. 2021 Dec 14;5(1):172–82. doi: 10.1021/acsabm.1c01027 (PMC8767544; doi:10.1021/acsabm.1c01027)
Supplement: Supplementary file 1 — mt1c01027_si_001.pdf [file mt1c01027_si_001.pdf]

SUPPORTING INFORMATION

FINITE ELEMENT ANALYSIS OF A BIONATE® RING-SHAPED CUSTOMIZED LUMBAR DISC NUCLEUS PROSTHESIS

Amparo Vanaclocha-Saiz[1], Vicente Vanaclocha* MD PhD[2], Carlos M. Atienza[3,4], Pablo Clavel MD[5], Pablo Jorda-Gomez MD[6], Carlos Barrios-Pitarque MD PhD[7], Leyre Vanaclocha MD[8]

[1]Escuela de Doctorado, Universitat Politècnica de Valencia, Camí de Vera, s/n, 46022 Valencia, Spain

[2]University of Valencia, Avenida de Blasco Ibáñez, 13, 46010 Valencia, Spain

[3]Instituto de Biomecánica (IBV), Universitat Politècnica de Valencia, Camí de Vera, s/n, 46022 Valencia. Spain

[4]Instituto de Biomecánica de Valencia-CIBER BBN, Grupo de Tecnología Sanitaria (GTS-IBV), Camí de Vera, s/n, 46022 Valencia, Spain

[5]Instituto Clavel, Hospital Quironsalud Barcelona, Plaça d'Alfonso Comín, 5, 08023 Barcelona, Spain

[6]Hospital Politècnic i Universitari La Fe, Avinguda de Fernando Abril Martorell, 106, 46026 Valencia, Spain

[7]Catholic University of Valencia, Saint Vincent Martyr, Carrer de Quevedo, 2, 46001 Valencia, Spain

[8]University College London, London, Gower St, London WC1E 6BT, United Kingdom

Corresponding author

Professor Vicente Vanaclocha

University of Valencia, Valencia, Spain

vivava@uv.es

Telephone +34 669 79 00 13, Fax + 34 96 340 99 22

SUPPLEMENTARY MATERIAL

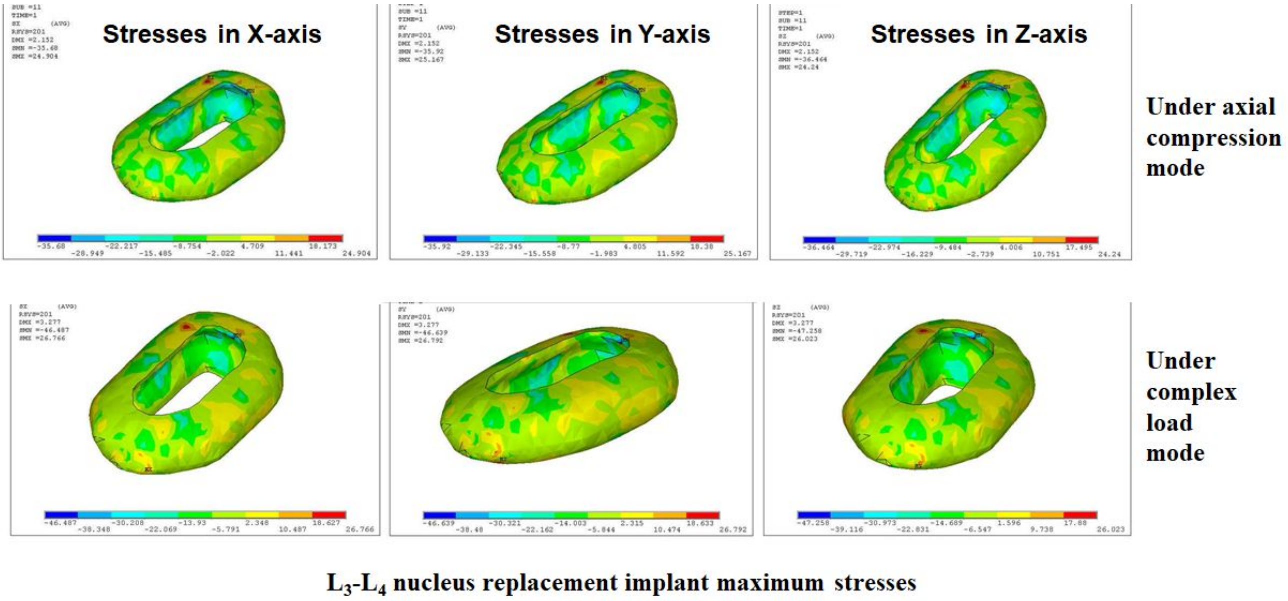

Figure 1S. L_3-L_4 Nucleus replacement implant maximum stresses under axial compression and complex load modes.

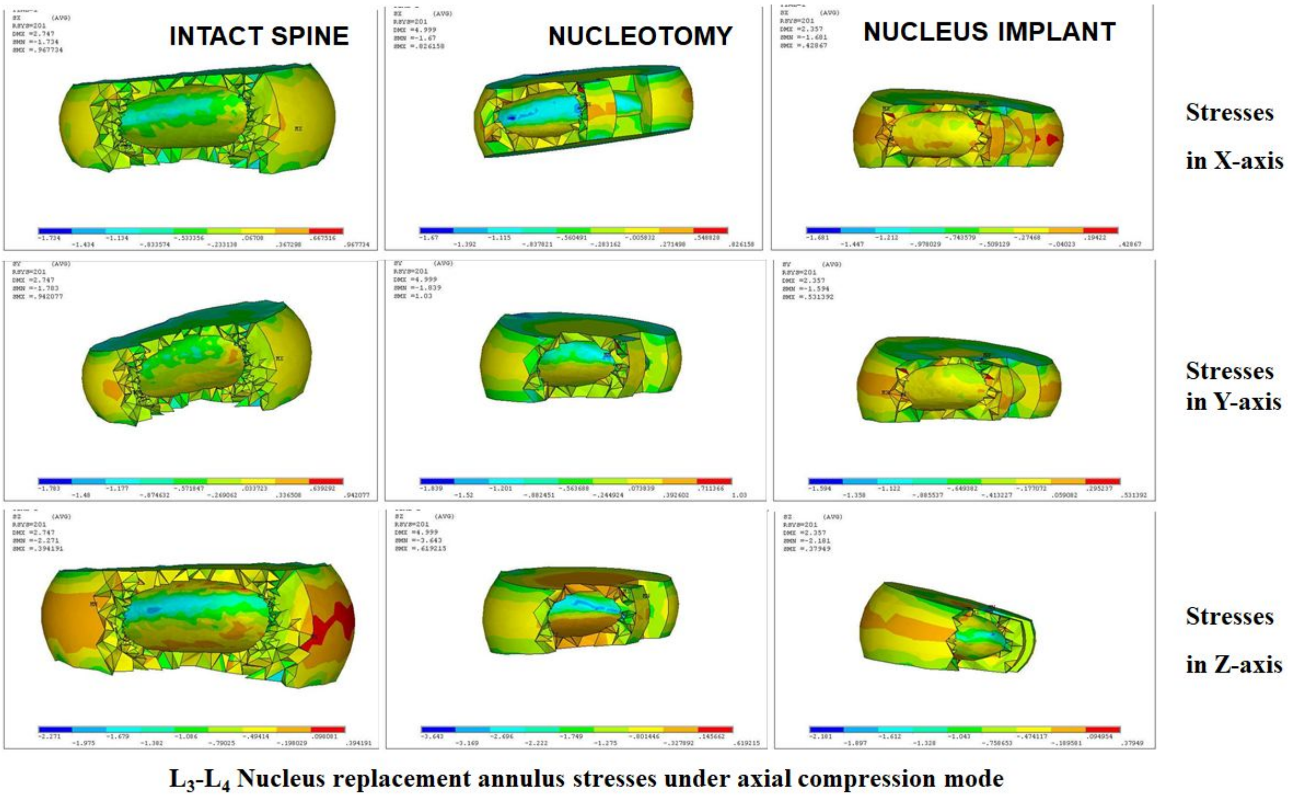

Figure 2S. L_3-L_4 Nucleus replacement annulus stresses under axial compression mode.

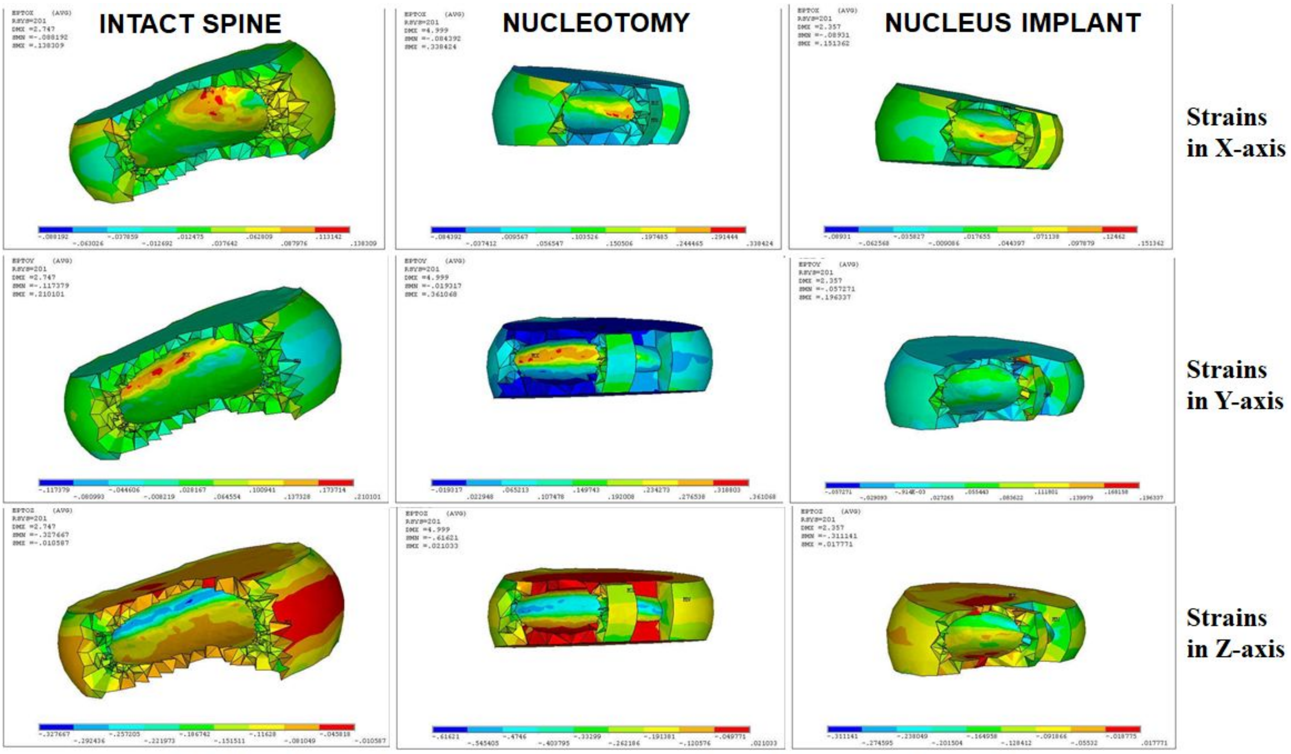

L_3-L_4 Nucleus replacement annulus strains under axial compression mode

Figure 3S. L_3-L_4 Nucleus replacement annulus strains under axial compression mode.

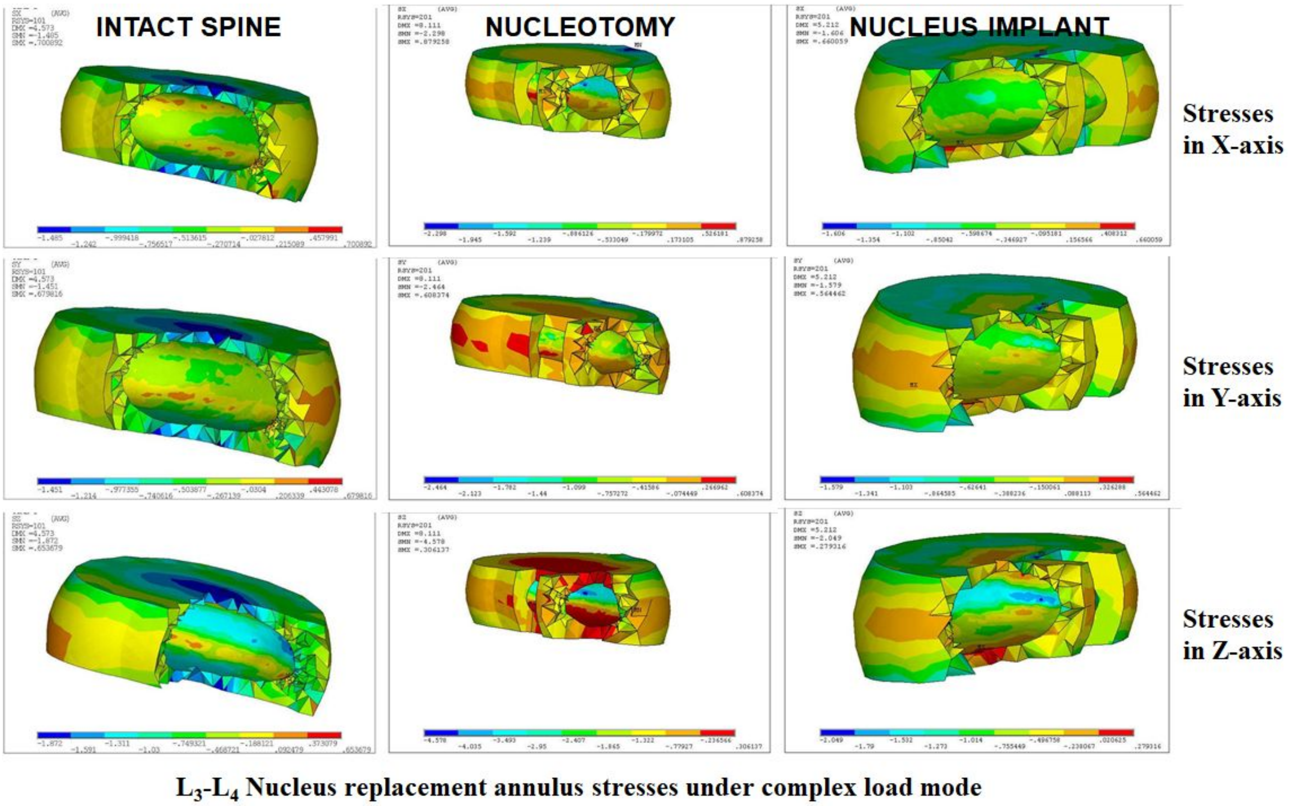

Figure 4S. L_3-L_4 Nucleus replacement annulus stresses under complex load mode.

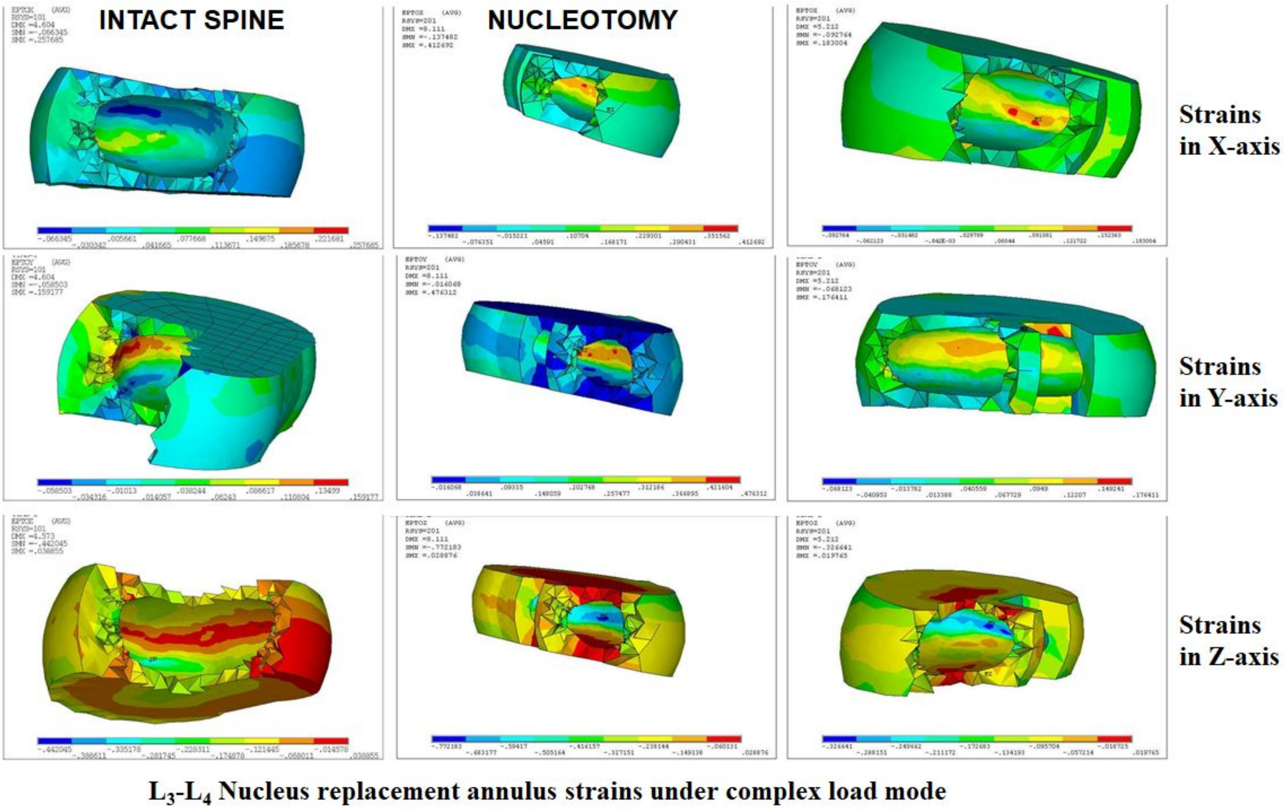

Figure 5S. L_3-L_4 Nucleus replacement annulus strains under complex load mode.

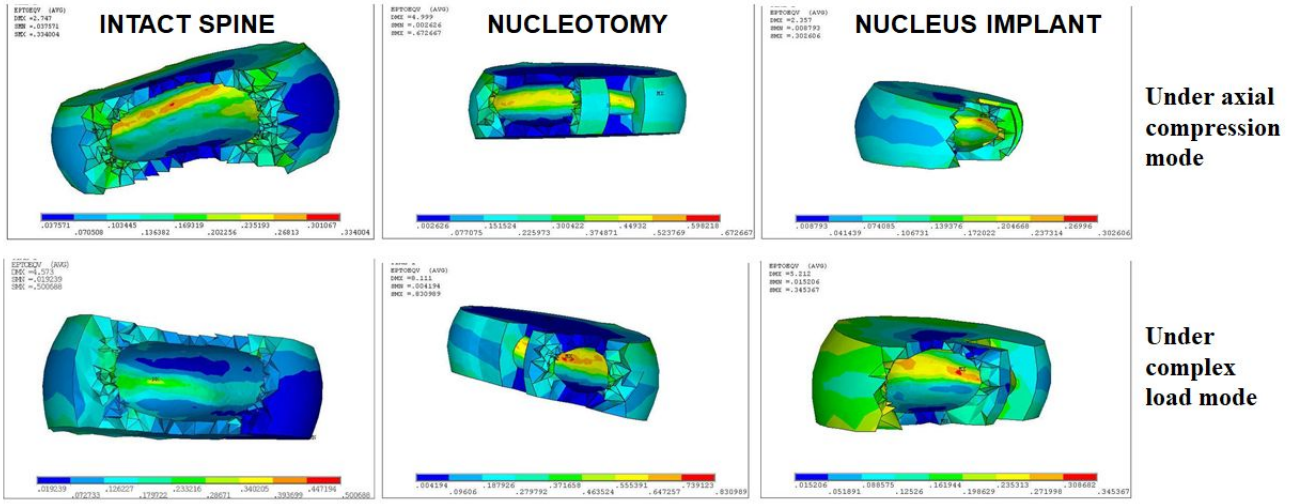

L_3-L_4 nucleus replacement annulus Von Misses strains

Figure 6S. L_3-L_4 Nucleus replacement annulus Von Misses strains under axial compression and complex load modes.

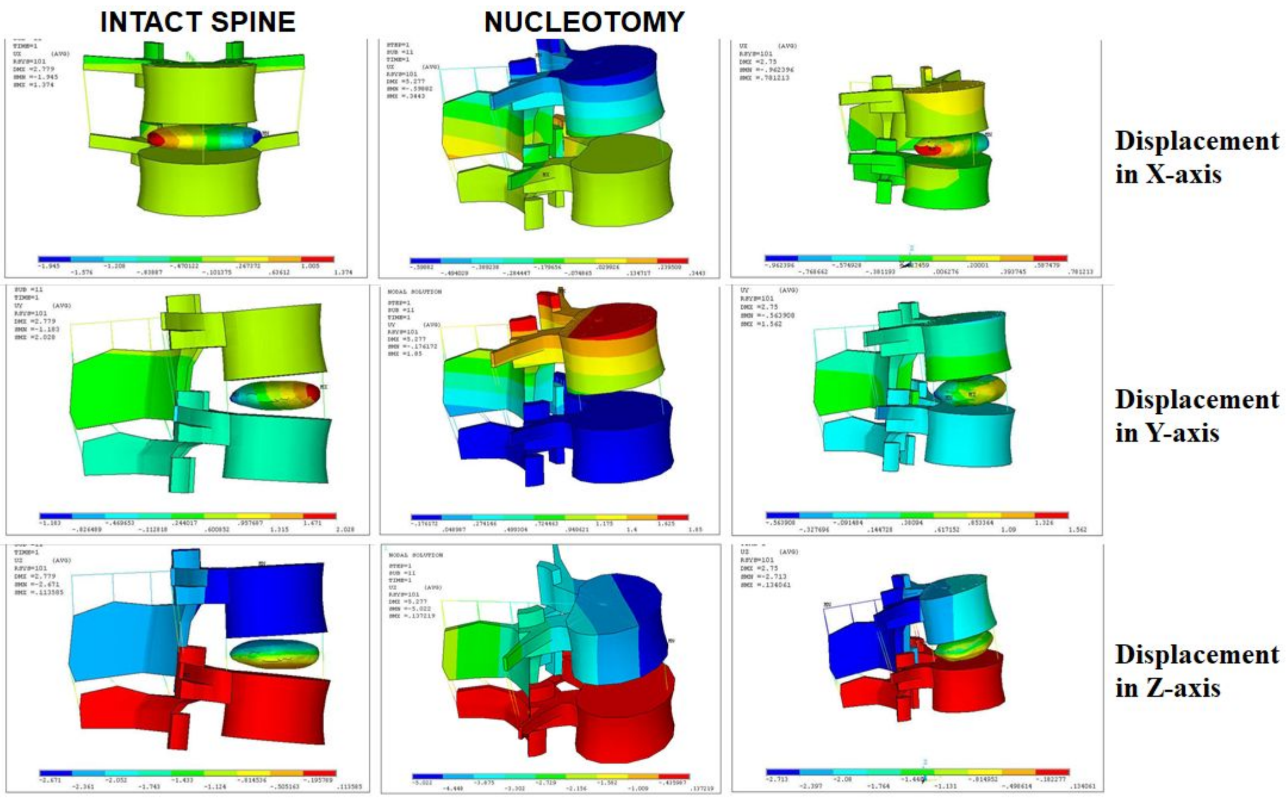

L_3-L_4 Nucleus replacement relative motion of vertebrae under axial compression mode

Figure 7S. L_3-L_4 Nucleus replacement relative motion of vertebrae under axial compression mode.

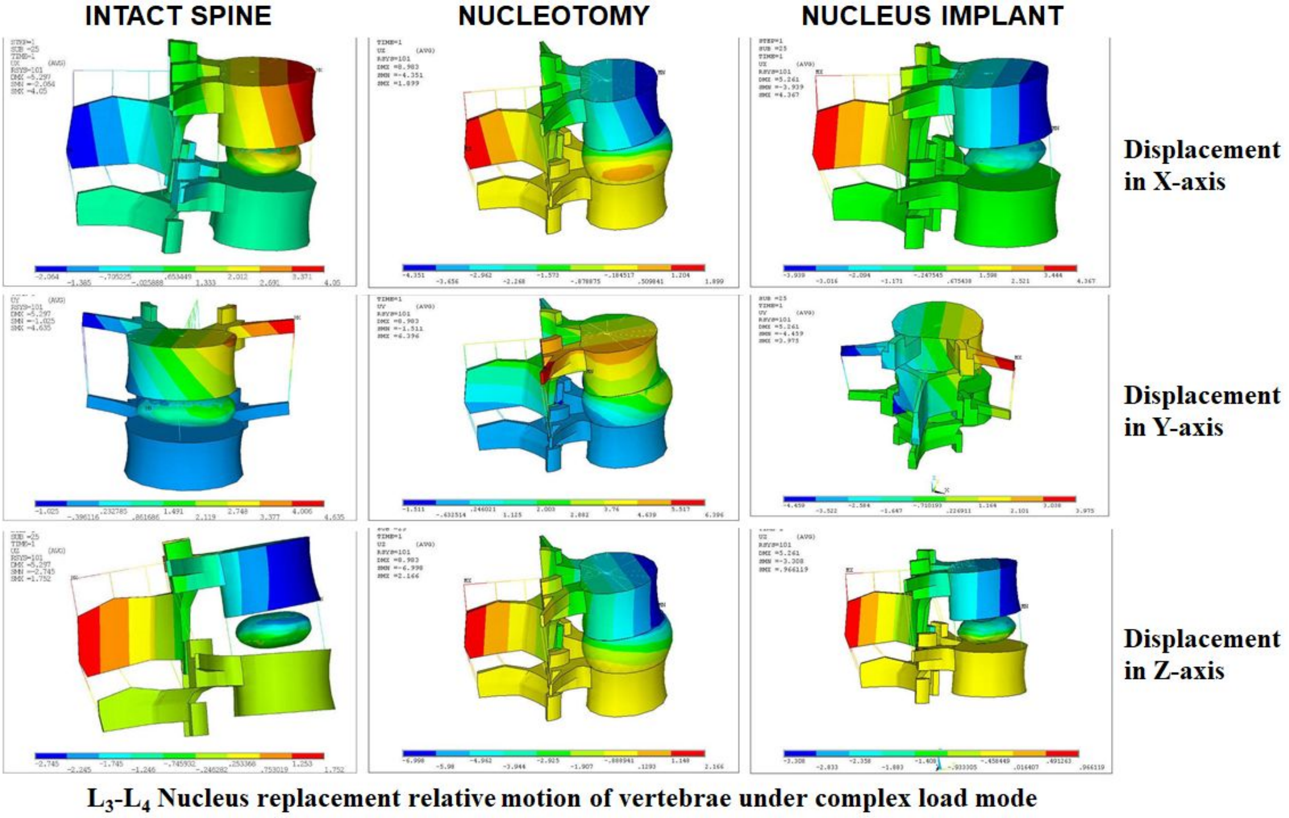

Figure 8S. L_3-L_4 Nucleus replacement relative motion of vertebrae under complex load mode.

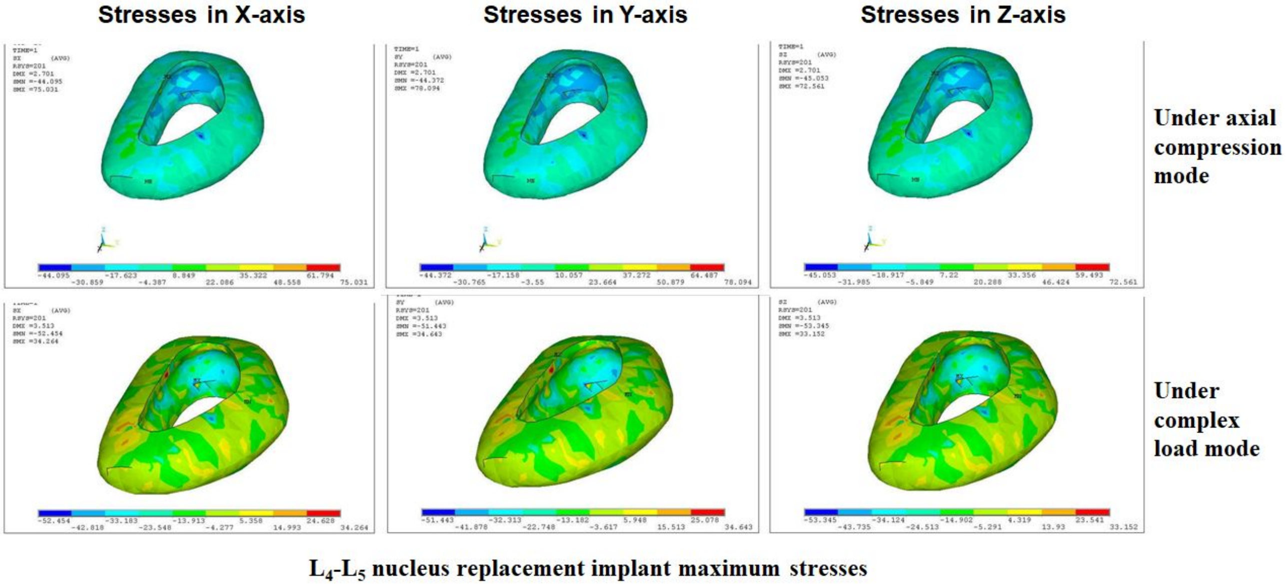

Figure 9S. L_4-L_5 Nucleus replacement implant maximum stresses under axial compression and complex load modes.

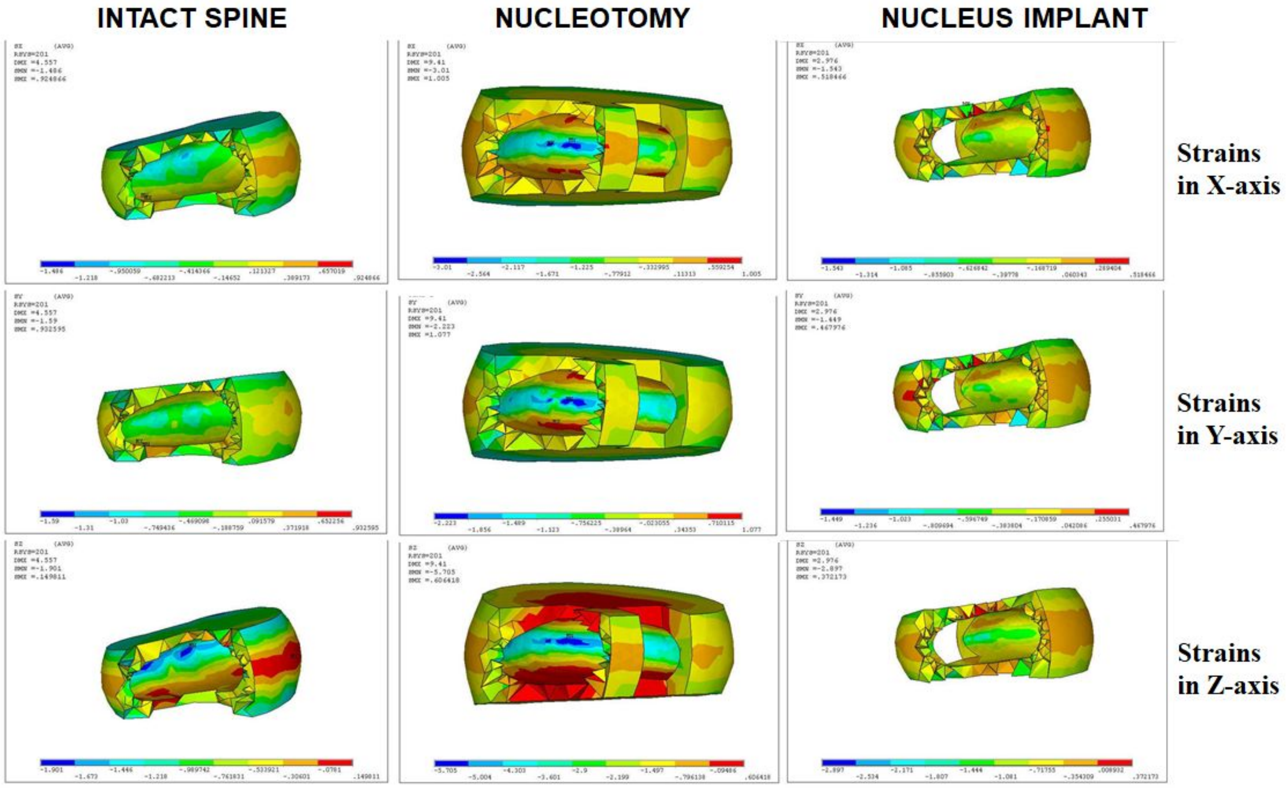

L_4-L_5 Nucleus replacement annulus stresses under axial compression mode

Figure 10S. L_4-L_5 Nucleus replacement annulus stresses under axial compression mode.

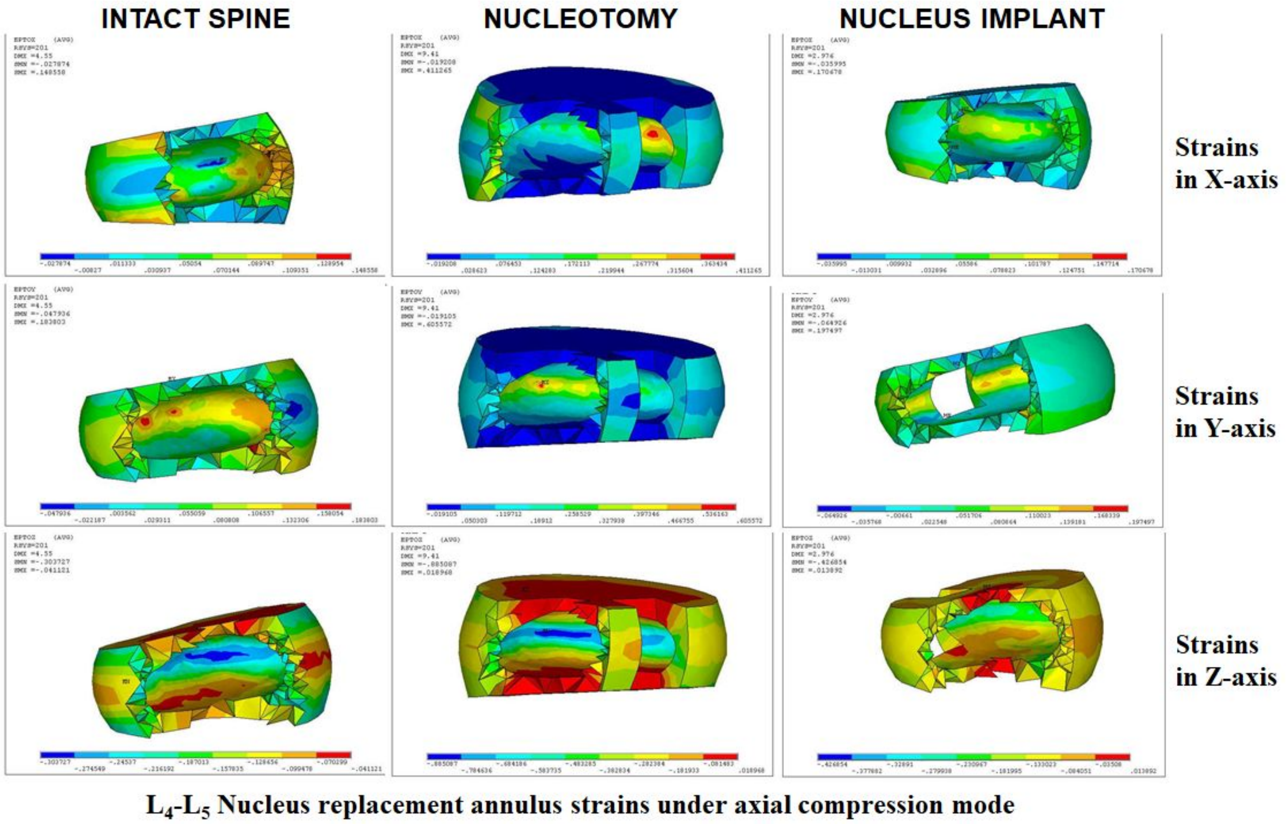

Figure 11S. L_4-L_5 Nucleus replacement annulus strains under axial compression mode.

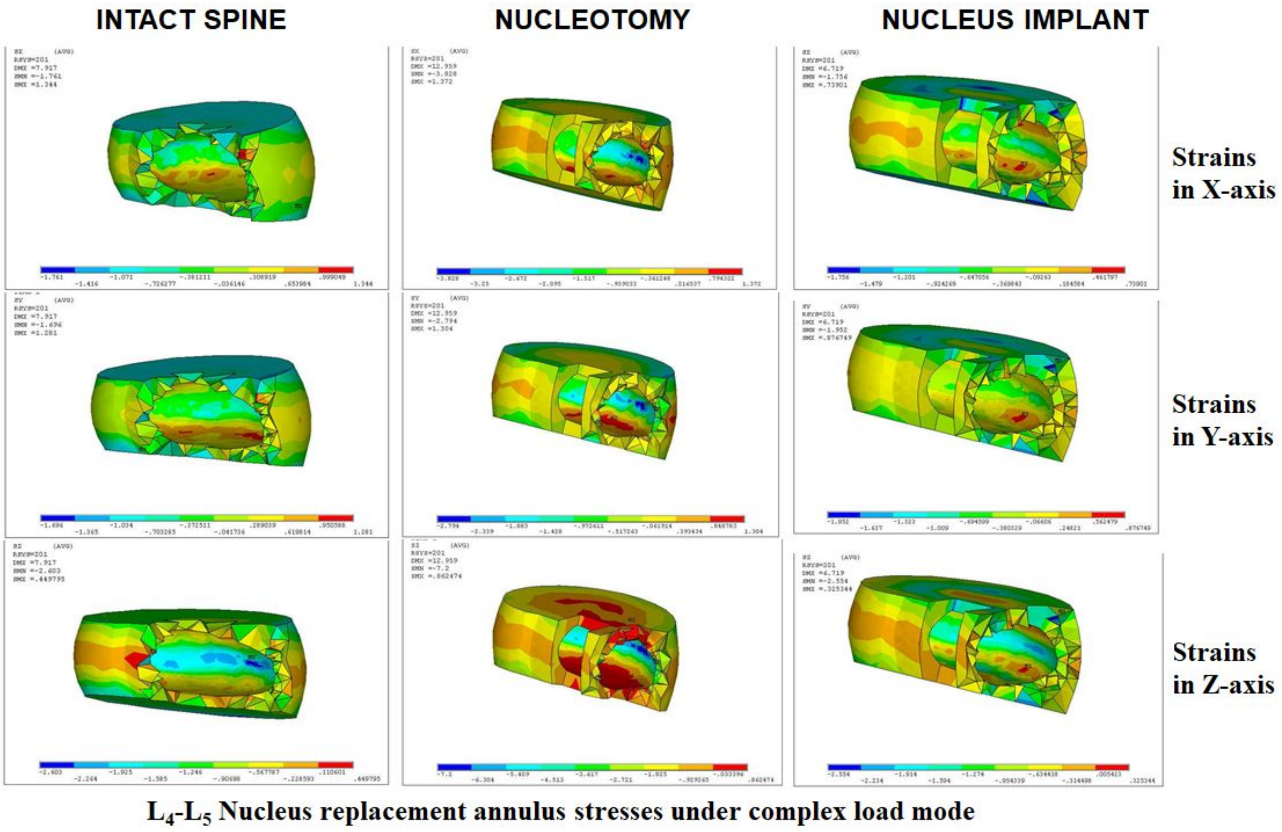

Figure 12S. L_4-L_5 Nucleus replacement annulus stresses under complex load mode.

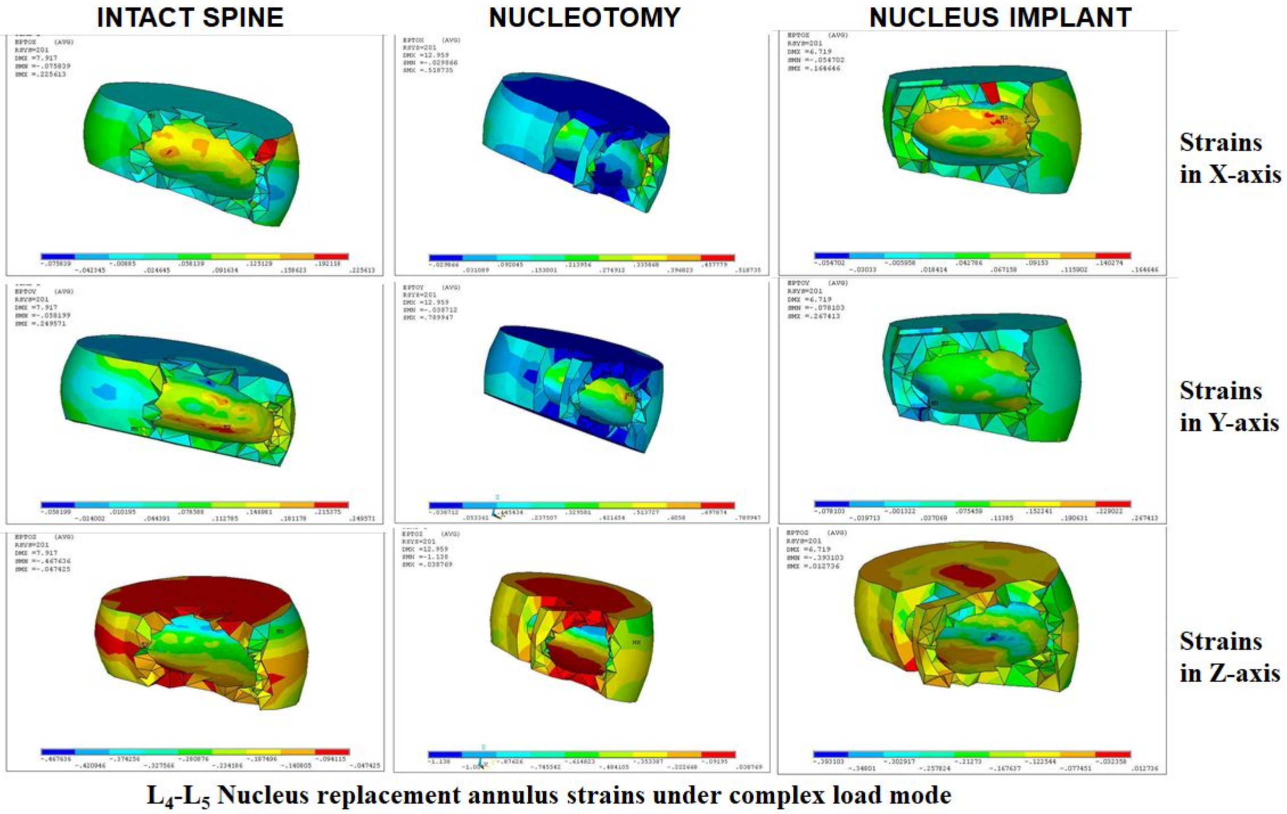

Figure 13S. L_4-L_5 Nucleus replacement annulus strains under complex load mode.

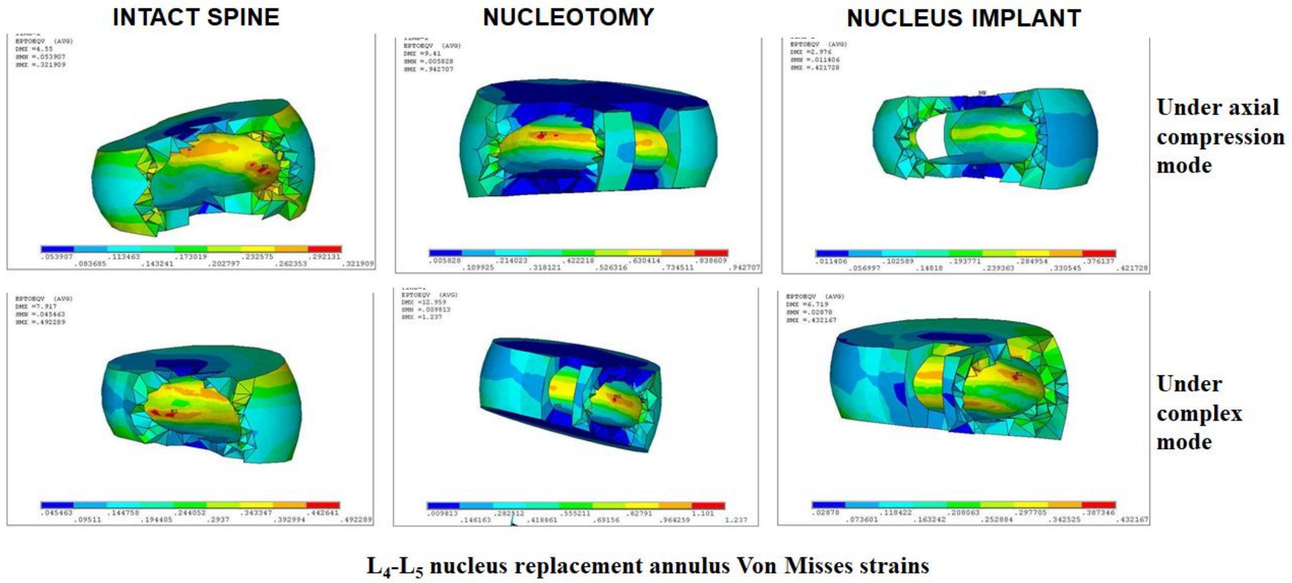

Figure 14S. L_4-L_5 Nucleus replacement annulus Von Misses strains under axial compression and complex load modes.

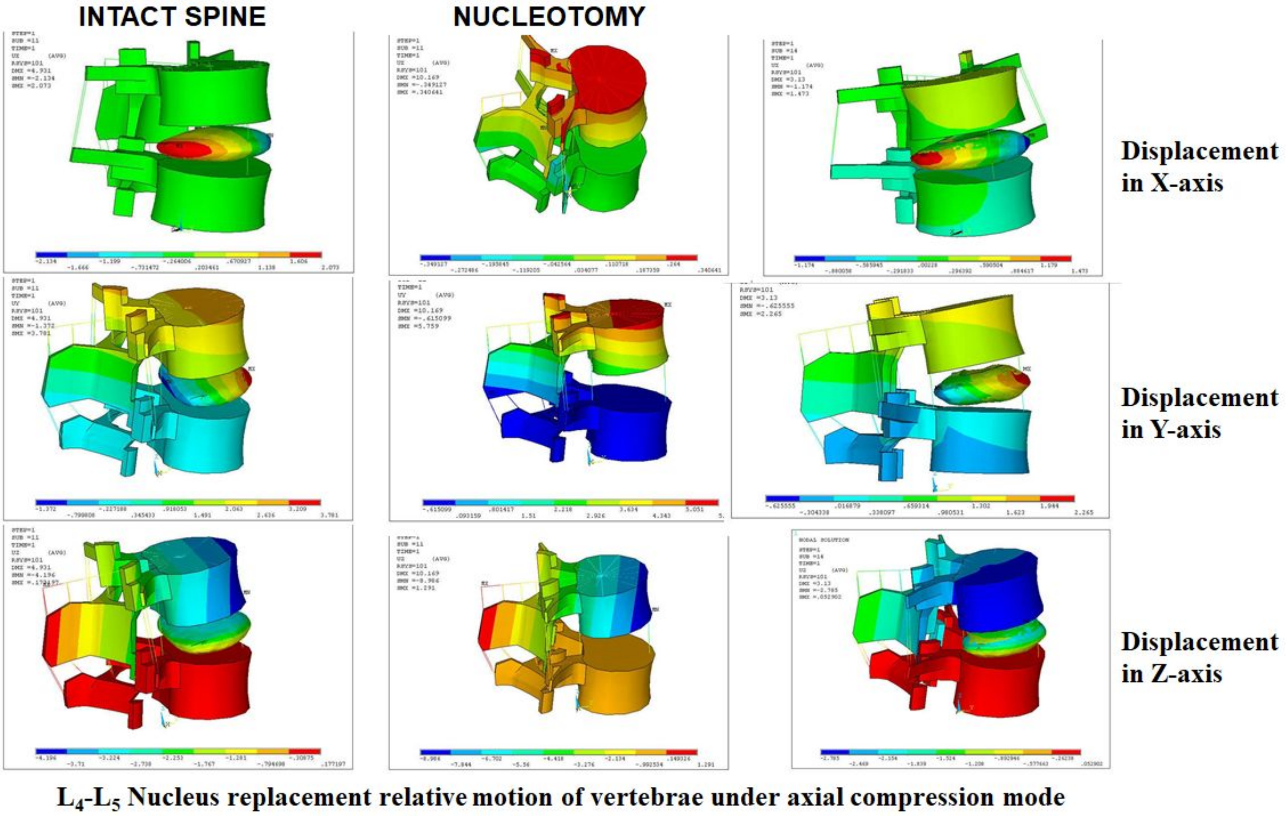

Figure 15S. L_4-L_5 Nucleus replacement relative motion of vertebrae under axial compression mode.

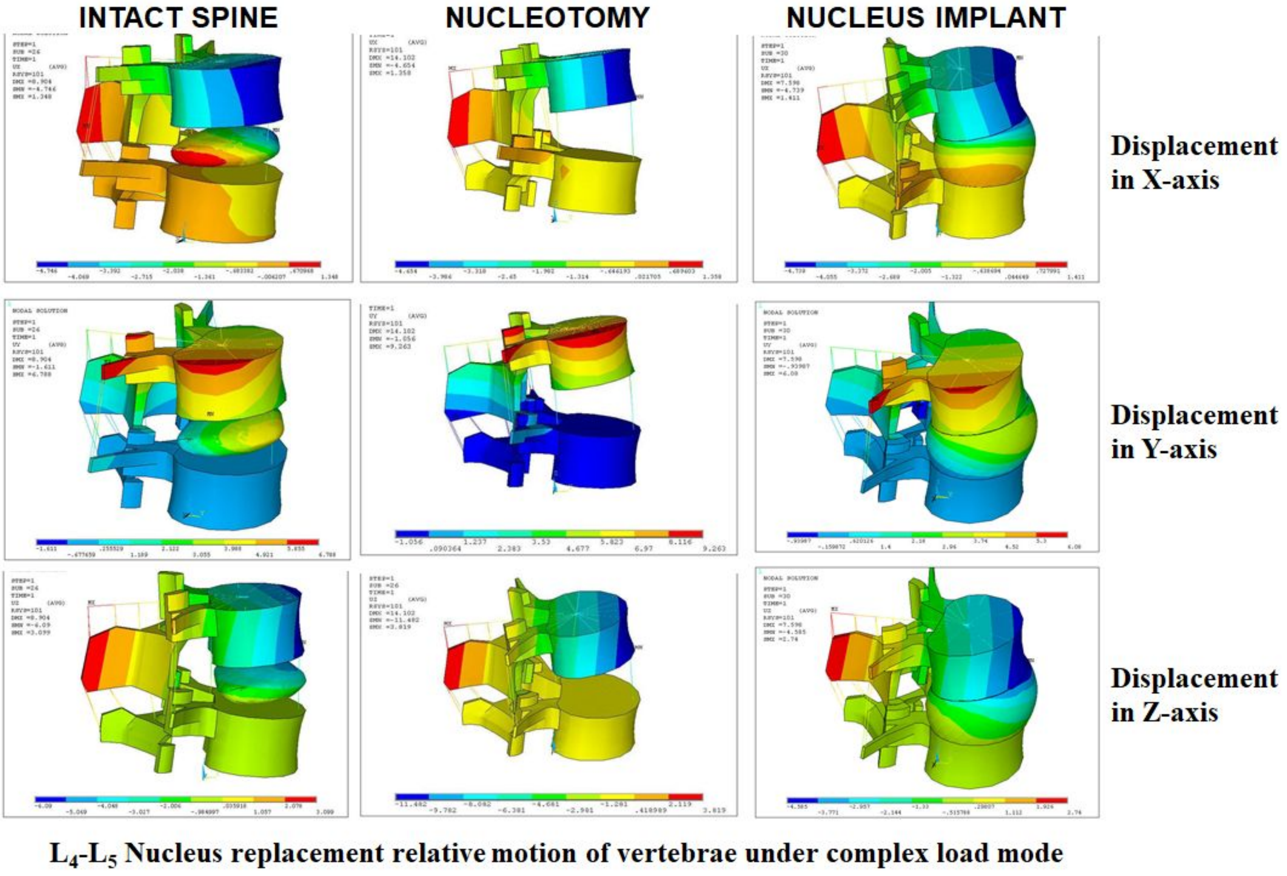

Figure 16S. L_4-L_5 Nucleus replacement relative motion of vertebrae under complex load mode.

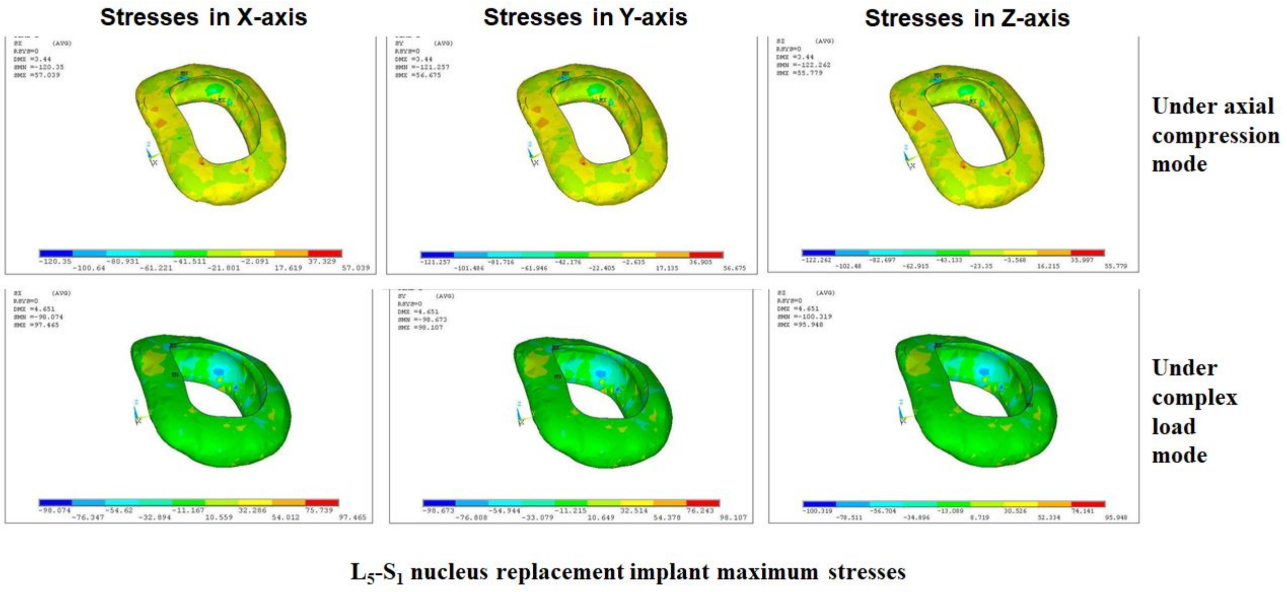

Figure 17S. L_5-S_1 Nucleus replacement implant maximum stresses under axial compression and complex load modes.

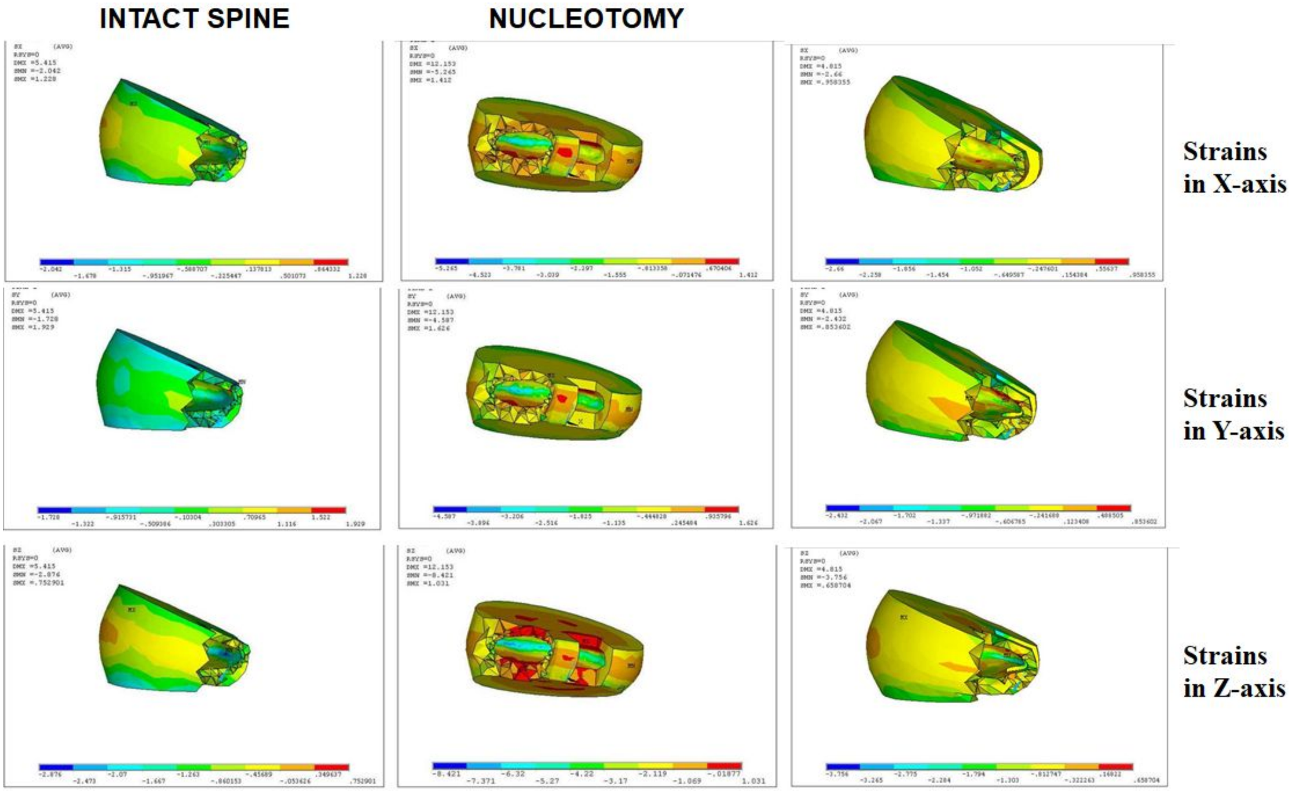

L_5-S_1 Nucleus replacement annulus stresses under axial compression mode

Figure 18S. L_5-S_1 Nucleus replacement annulus stresses under axial compression mode.

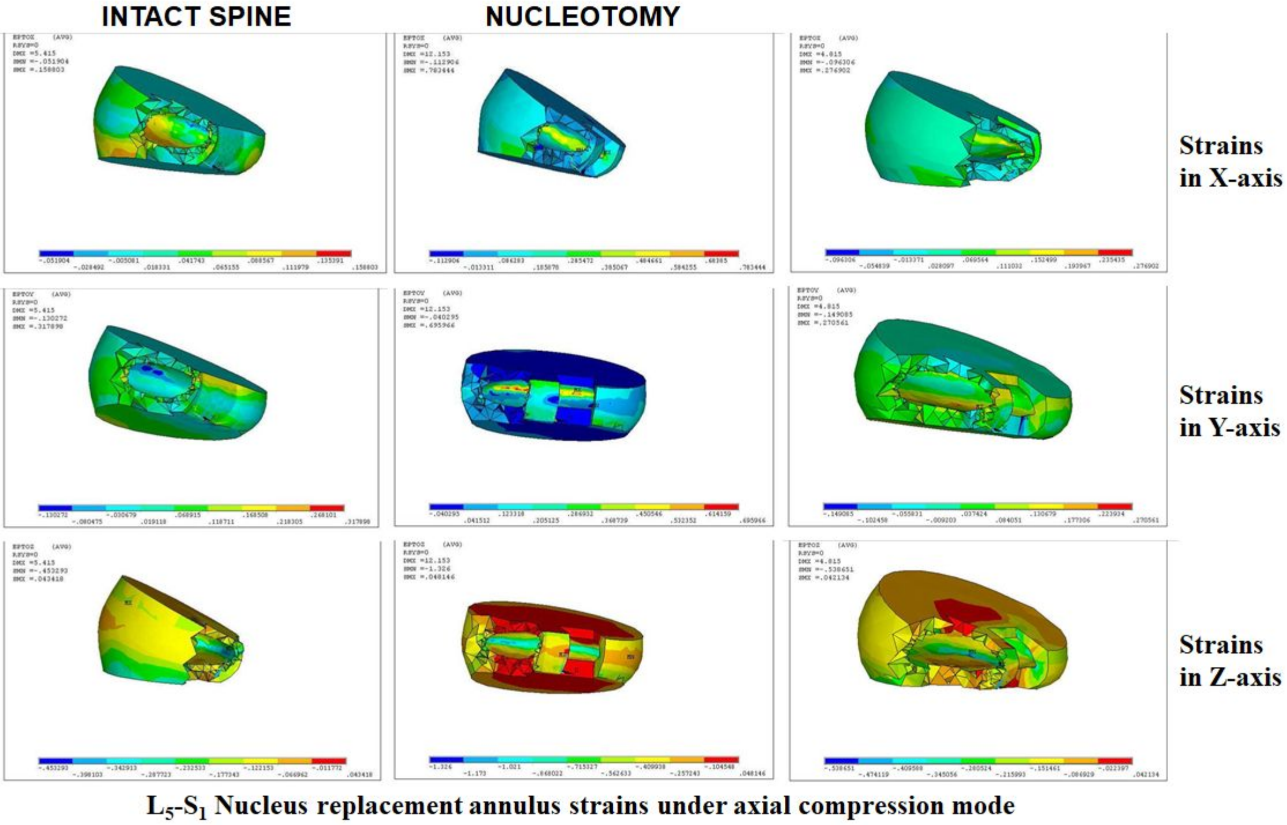

Figure 19S. L_5-S_1 Nucleus replacement annulus strains under axial compression mode.

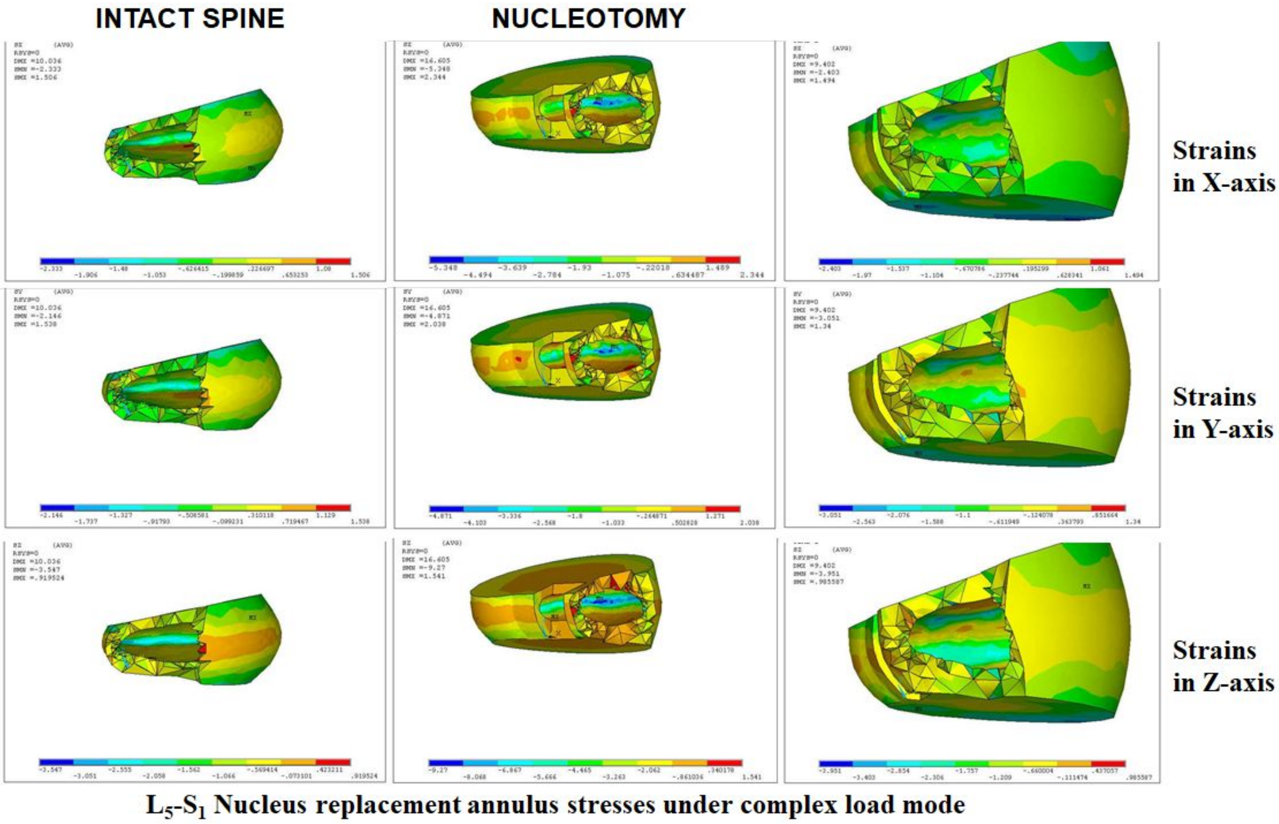

Figure 20S. L_5-S_1 Nucleus replacement annulus stresses under complex load mode.

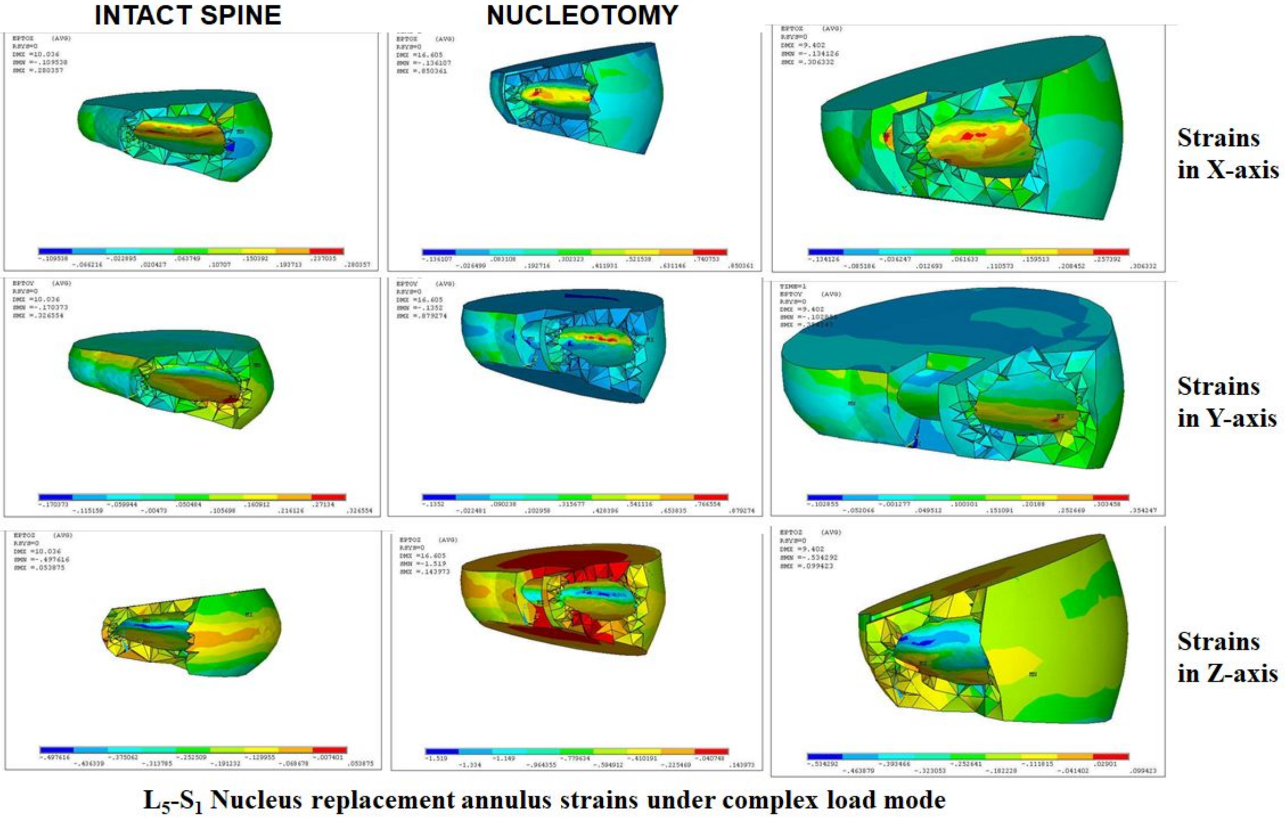

Figure 21S. L_5-S_1 Nucleus replacement annulus strains under complex load mode.

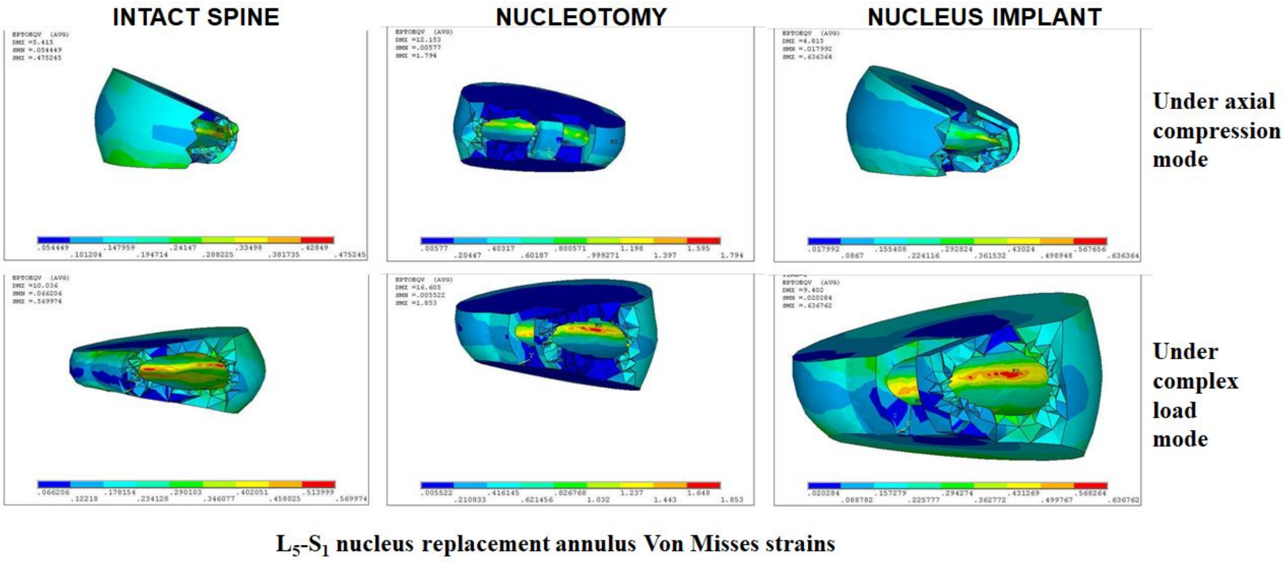

Figure 22S. L_5-S_1 Nucleus replacement annulus Von Misses strains under axial compression and complex load modes.

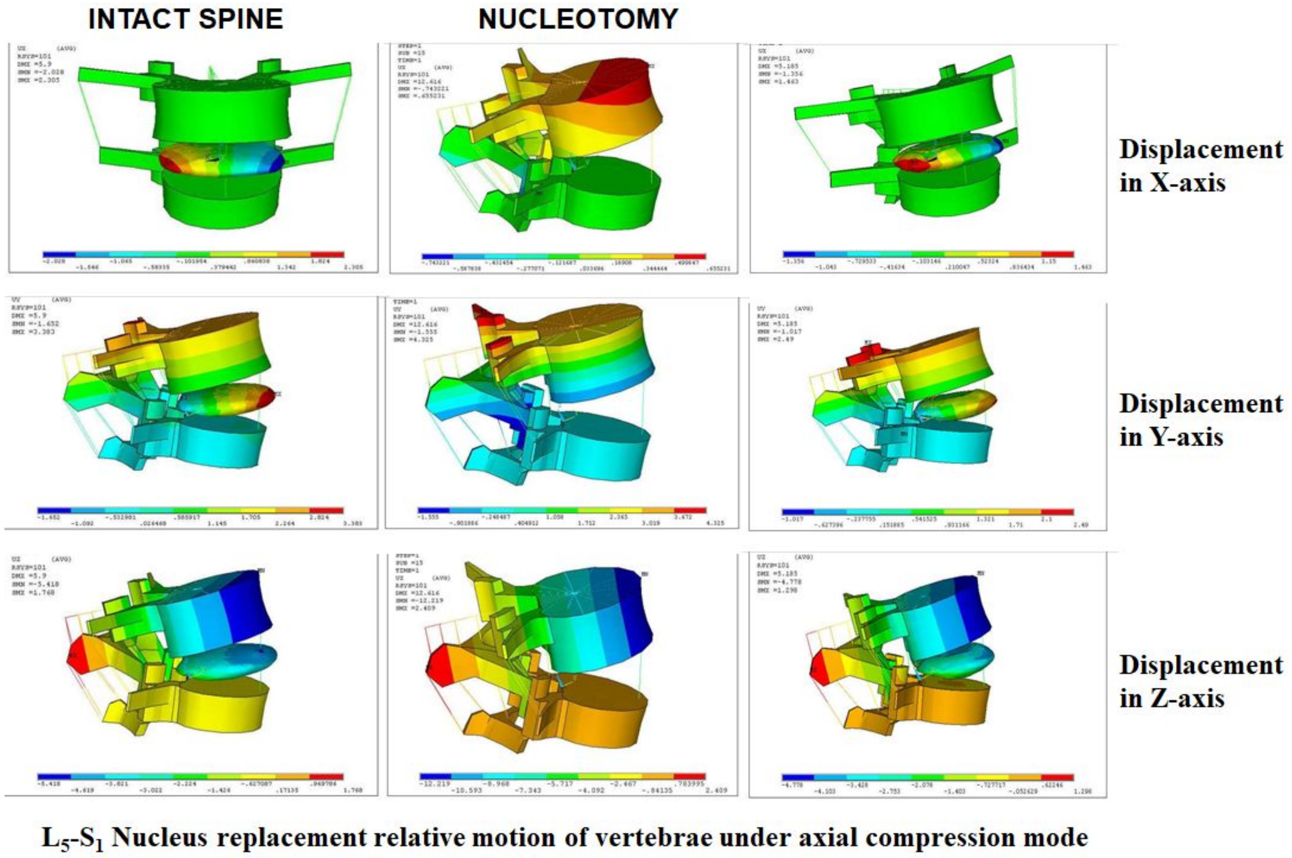

Figure 23S. L_5-S_1 Nucleus replacement relative motion of vertebrae under axial compression mode.

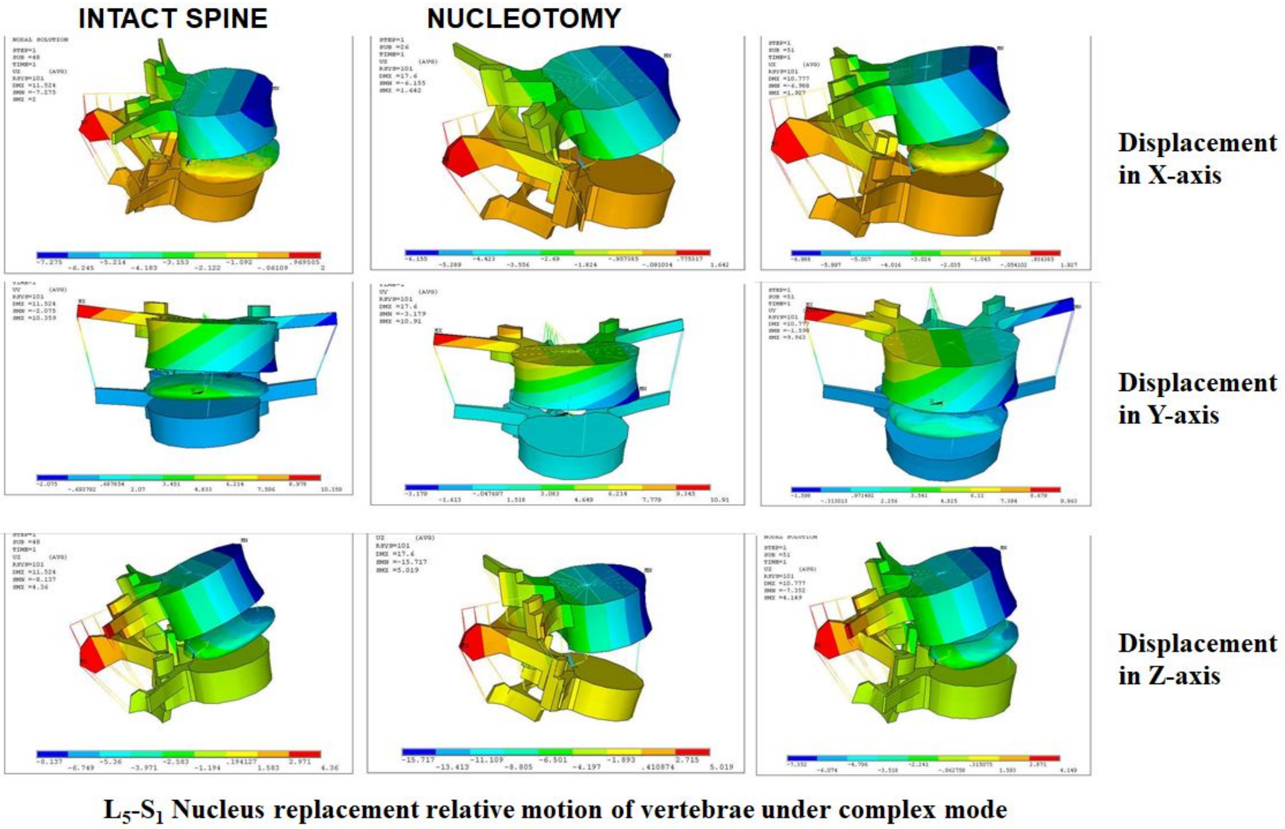

Figure 24S. L_5-S_1 Nucleus replacement relative motion of vertebrae under complex load mode.
